# Can We Merge the Weak and Strong Tetrel Bonds? Electronic Features of Tetrahedral Molecules Interacted with Halide Anions

**DOI:** 10.3390/molecules27175411

**Published:** 2022-08-24

**Authors:** Ekaterina V. Bartashevich, Svetlana E. Mukhitdinova, Iliya V. Klyuev, Vladimir G. Tsirelson

**Affiliations:** 1 Chemistry Department, South Ural State University (National Research University), 76, Lenin Av., 454080 Chelyabinsk, Russia; 2 Quantum Chemistry Department, D.I. Mendeleev University of Chemical Technology, 125047 Moscow, Russia

**Keywords:** tetrel bond, orbital-free quantum crystallography, electron density, total one-electron static potential, electrostatic potential, Pauli potential

## Abstract

Using the orbital-free quantum crystallography approach, we have disclosed the quantitative trends in electronic features for bonds of different strengths formed by tetrel (Tt) atoms in stable molecular complexes consisting of electrically neutral tetrahedral molecules and halide anions. We have revealed the role of the electrostatic and exchange-correlation components of the total one-electron static potential that are determined by the equilibrium atomic structure and by kinetic Pauli potential, which reflects the spin-dependent electron motion features of the weak and strong bonds. The gap between the extreme positions in the electrostatic and total static potentials along the line linking the Tt atom and halide anion is wide for weak bonds and narrow for strong ones. It is in very good agreement with the number of minima in the Pauli potential between the bounded atoms. This gap exponentially correlates with the exchange-correlation potential in various series with a fixed nucleophilic fragment. A criterion for categorizing the noncovalent tetrel bonds (TtB) based on the potential features is suggested.

## 1. Introduction

### 1.1. Tetrel Bonds

The aim to understand the nature of various chemical bonds and their categorization reflects the global challenges and trends in theoretical chemistry and computational material science. The categorization of noncovalent bonds is based on the ability of the elements belonging to Groups 14–17 of the periodic table (PT) to provide the local electrophilic region in the inhomogeneous electron shells and to define the name of the specific bond [1,2,3]. This systematization stands on the concept of the anisotropy of electron density in the valence shell of a covalently bound atom, which leads to the formation of the σ- and π-holes [4]. This effect is easily observable and this is one of the reasons why corresponding interactions are called “electrostatically driven” ones [5]. Nowadays, several important discussions have taken place, and, as a result, noncovalent bonds acquired the following names: *halogen bond*, XB (atom X of Group 17 of PT provides its electrophilic region for noncovalent bonding); *chalcogen bond*, ChB (atom Ch of Group 16); *pnictogen bond*, PnB (atom Pn of Group 15); *tetrel bond*, TtB (atom Tt of Group 14). By the analogy with IUPAC definitions [6,7] formulated for “-*ogen bonds*”, a tetrel bond could be defined as an attractive interaction between a local electrophilic region of an atom belonging to Group 14 of PT with a nucleophilic region in the same or another molecule.

Tetrel bonds are now intensively studied from both experimental and theoretic points of view [1]. The elements of the carbon group are part of the most important compounds of reinforcing fillers, carbon fibers, nanotubes, and nanoparticles, so the issues of their involvement in noncovalent interactions deserve special attention. Several features significant for the practice of TtB formation by carbon materials have been mentioned in the review [8]. Computational modeling has shown [9] that the strength of TtB increases in a row C < Si < Ge. Heavier tetrels can form stronger TtB than carbon because of their higher polarizability.

Analysis of computational spectral data used for the estimation of the TtB strength [10] has shown that it is impossible to specify the sole determining factor, and we should consider several of them simultaneously. Firstly, the bonding is stronger with the increase in the atomic number of the Tt-atom and its polarizability. Secondly, the strongest bond is formed with halogen substituents. Thirdly, the Tt-atom involved in a double bond forms a weaker interaction. The correlation between the frequency shift in the IR spectra, chemical shielding in NMR and parameters of noncovalent bonds from halogen to tetrel, including the complexes of the FH_3_Tt molecules, where Tt = Si, Ge, Sn, have been studied [11]. It has been shown that the highest frequency shift is observed for XB, while the lowest one is for PnB. In NMR, the changes in shielding are decreased from XB to TtB, but for the donor of the electrons, they are the highest for TtB. It has been suggested [12] that the X_2_TtO molecules (X = H, F, Cl, Br, CH_3_; Tt = C, Si, Ge, Sn) can act as effective adsorbents of CO_2_ due to the TtB formation. The majority of complexes with CO_2_ are stabilized by the combination of C…O and O…Tt interactions and their energy of interaction increases in a row C < Ge < Sn < Si.

For molecular complexes, the ability of the TtB formation with the massive nucleophile atoms is higher for heavier representatives of Group 14. Steric effects preventing a binding could be compensated by the increase of the Tt-atom size by the addition of electron-acceptor substituents and by the strengthening nucleophiles such as anions. The energy of TtB reaches up to 10 kcal/mol for the pair of neutral molecules without an electron-acceptor substituent: the addition of CF_3_-group increases the energy of TtB and attains its maximum of almost 54 kcal/mol in anionic complexes [13].

The results of diverse studies of the geometries, energy, and electronic characteristics of TtBs, formed by heavy atoms, such as Sn and Pb, are summarized in [14]. The stabilization of the complexes formed by σ- and π-hole interactions, especially for specific anion binding, is discussed in [15,16,17]. The reactivity of the gas–phase complexes in the S_N__2_ reactions is actively studied [18]. It has been demonstrated that the SnF_3_ group is able to form strong bonds with anions (~50 kcal/mol) and can be used for the extraction of target anionic pollutants from water solutions. The specific binding via S…Sn interactions has been considered using structural data from the Protein Data Bank with further docking of organotin compounds into the active center of the receptor [19]. It has been observed that the formation of S…Sn interactions reinforced by the assisted CH…π interaction takes place and it promotes the receptor activation controlling further gene expressions. 

A rigorous analysis of CSD data for TtBs [20], formed by the C(sp^3^) atom, which is bound to cationic (ammonium, sulfonium) and neutral (F и NO_2_) substituents, has been recently performed in the framework of crystal design methodology. It has been revealed that the linear direction of R–C…B interactions is an important characteristic of TtBs. The ability to form TtB is also treated as a tool for crystal engineering. A mention has recently been made [21] about the lack of experimental data concerning the interactions of Ge and Sn with nucleophiles. For several crystalline systems with fragments Hal^−^…CH_3_–Y (Hal^−^ = Cl, Br; Y = N, O) it has been confirmed that the carbon atom in the methyl group presents its electrophilic region in a typical TtB [22]. The electronic criterion [23] based on the disposition of the minima of electron density and electrostatic potential along the TtB line has been applied.

However, the analysis of CSD shows the existence of numerous short contacts between Ge and Sn with the atoms bearing electron lone pairs and reveals that such interactions can define the conformations and packing of the organometallic crystals. Analysis of experimental short-contact data from CSD for the crystalline complexes of Pb and Sn [21,24] has led to the conclusion that TtBs formed by Sn do not only widely take place in a solid state, but also promote the stabilization of complexes in a crystal form. Intramolecular TtBs Sn…X (X=O, S, N, F, Cl, Br, I) also play a significant role in the stabilization of the preferable conformation. There are examples of TtBs, formed by Pb(II) [24], which could be used in the tasks of crystal engineering for the formation of 2D and 3D motives and the generation of the crystal packing of the organometallic framework. The usage of SnPh_3_ chloride as a donor of TtB [25] has allowed for 10 different co-crystals with short, as well as linear TtBs, to be obtained, and such a donor can be used for the crystal engineering of packing with a predefined topology of bonds.

The idea to use the local electronic characteristics, P(r_bcp_), at the bond critical points (bcp) of electron density in the analysis of chemical bonding came from QTAIM [26,27,28]. Bond critical points (if they exist) are the specific points corresponding to the minimum of electron density along the interatomic line and to its maxima in the orthogonal directions. For evaluating the energy of hydrogen bonds, EHB, the single-factor correlation models were suggested, such as the models “E_HB_ vs. P(r_cp_)”, where P(r_cp_) was the potential v(r_bcp_), or kinetic g(r_bcp_) electronic energy density [29,30,31,32,33]. Further, that idea was extended to different types of noncovalent bonds [34,35,36,37]. The electrostatic potential at bcp was used as a factor in correlation models [38,39]. It was found [40] that those different principles of sorting noncovalent bonds in a sample led to the superiority of some factors over others. The choice of single-factor models depends on the fixation or variation of the atom donating its local electrophilic region for noncovalent bonding. Simultaneously, the fixation/variation of a nucleophilic fragment affects the behavior of linear trends. Therefore, while estimating the energy of a bond from parametric equations and making predictions, it is important to select the appropriate parameters of the model.

### 1.2. Orbital-Free Quantum Crystallography Approach

Orbital-free density functional theory [41] allows extending QTAIM and joins it with the quantum crystallography [42,43]. It starts from the one-electron Euler equation

(1) $$\mu \left(r\right)=\frac{{\delta T}_{s}\left[\rho \right]}{\delta \rho }+\upsilon_{\mathrm{st}}\left(r\right)$$

which connects the static one-electron potential, υ_st_(**r**), and the functional derivative of the noninteracting kinetic energy of N electrons, T_s_[ρ], with respect to the electron density. A Lagrange multiplier µ, an electronic chemical potential, follows from the condition ∫ρ(**r**)d**r** = N. A static potential

(2) $$\upsilon_{\mathrm{st}}\left(r\right)=-\upsilon_{\mathrm{es}}\left(r\right)+\upsilon_{\mathrm{xc}}\left(r\right)$$

includes the electrostatic (es) and exchange-correlation (xc) components that depend on the equilibrium nuclear configuration and on the corresponding electron density. It often appears in the literature as a potential acting on an electron in a molecule [44,45,46]. In a single-determinant approximation, T_s_[ρ] is presented as a sum of the contributions:

(3) $$T_{s}\left[\rho \right]=T_{W}\left[\rho \right]+T_{P}\left[\rho \right]$$

The Weizsäcker (1935) kinetic energy $T_{W}\left[\rho \right]=\int^{\;}\frac{{\left|\nabla \rho \left(r\right)\right|}^{2}}{8\rho \left(r\right)}dr$ > 0 originates from the wave–particle duality of electrons and an uncertainty principle [47]. It arises from a semi-local part of the quantum electron fluctuations and presents the kinetic energy of noninteracting “spinless” particles of density ρ(**r**), which are in the hypothetical “bosonic” ground state, where all particles are in the same lowest energy state [48]. However, the presence of an electron of a given spin at **r** affects the motion of the rest of the same-spin electrons near this point. Therefore, to fit the antisymmetry requirement for the many-electron wavefunction, the Pauli energy [49,50], T_P_[ρ], is introduced. It describes the excess in the total electronic kinetic energy over the energy of the noninteracting “spinless” particles. 

Taking the functional derivative of T_s_[ρ] and supposing the proper homogeneity of the functionals in density scaling, the Euler equation for the stationary state is re-written via corresponding potentials as

(4) $$\mu =\upsilon_{\mathrm{kin}}\left(r\right)+\upsilon_{\mathrm{st}\;}{\left(r\right)}_{\;}$$

Here, the kinetic potential $\upsilon_{\mathrm{kin}}\left(r\right)=\upsilon_{P}\left(r\right)+\upsilon_{W}\left(r\right)$ contains the terms related to electron motion. 

All terms in (3) have clear physical and chemical meanings. They determine the one-electron homotropic and heterotropic forces [42] acting in a molecule and have been computed and implemented to the studies of chemical bonds in different compounds [51,52,53,54]. The Equations (2) and (3) lead to following expression for the Pauli potential [55,56,57].

(5) $$\upsilon_{P}\left(r\right)={\;\upsilon }_{\mathrm{es}}\left(r\right)-\upsilon_{\mathrm{xc}}\left(r\right)-\upsilon_{W}\left(r\right)+\mu$$

The electrostatic potential, υ_es_(**r**), is linked to the total (electron plus nuclear) charge density by the Poisson equation; there are approximations for the exchange potential, υ_xc_(**r**), and the Weizsäcker potential, υ_W_(**r**), is read as [49,58]

(6) $$\upsilon_{W}\left(r\right)=\frac{1}{8}\frac{{\left|\nabla \rho \left(r\right)\right|}^{2}}{\rho^{2}\left(r\right)}-\frac{1}{4}\frac{\nabla^{2}\rho \left(r\right)}{\rho \left(r\right)}$$

The outlined approach highlights physically significant components in the electron density and one-electron potentials connected with electrostatics, exchange, and spin-independent wave properties of an electron in a crystal. Electronic descriptors designed on the basis of this approach have been successfully tested for the analysis of the properties of chemical bonds in crystals [59]. In particular, for the halogen bonds the use of the electronic criterion has been suggested in order to distinguish electrostatically-driven noncovalent bonds from the weaker van der Waals interactions [60]. We will apply this methodology in our study of the electronic properties for a series of TtBs in molecular complexes to understand the nature of similarity and difference between weak and strong interatomic interactions in which the Tt atom participates as the provider of the electrophilic region. We have tried to answer the following specific questions.

- What electronic features of TtBs formed by tetrahedral molecules, where Tt = C, Si, Ge, Sn, Pb, might be made visible?
- How does the behavior of the Pauli potential, and electrostatic and static potentials differ for weak and strong bonds formed by a Tt atom?
- Could the exchange-correlation contribution to the static potential characterize TtB quantitatively?
- Is there a visible change in the properties at the junction of weak and strong TtBs? Can we merge them?

## 2. Materials and Methods

The equilibrium state modeling has been carried out for the complexes of halide anions, Hal = F^−^, Cl^−^, Br^−^, with electrically neutral tetrahedral molecules, Y–TtX_3_, Tt = C, Si, Ge, Sn, Pb; X = Cl, Br, CH_3_; Y = Hal, CH_3_, CN, NH_2_, NO_2_. In order to make the sample large and representative, we considered a halide anion as a nucleophile to avoid alternative binding with the rest of functional groups. In such complexes, the halide anion acts as the nucleophilic moiety, and the Tt atom of tetrahedral molecule Y–TtX_3_ delivers an electrophilic site. The geometry optimization of molecular complexes has been performed by Firefly software (v. 8.2.0), [61,62]. We have used the PBE0 [63] functional with Jorge-DZP-DKH [64,65,66] basis set from the Basis Set Exchange site [67]. Gradient convergence was 0.9 × 10^−6^. The optimized structures have been tested for the absence of imaginary IR frequencies.

The binding energy of complexes, E_bind_, has been estimated as the difference of the total energy of the optimized complex, E_AB_, and the sum of energies for isolated components: E_bind_ = E_AB_ − (E_A_ + E_B_) − E_CP_, see Table S3 in Supplementary Materials. Thus, E_bind_ describes the energy gain for tetrahedral molecule Y–TtX_3_ gathered in a complex with a halide anion. The BSSE correction, E_CP_, has been estimated, taking into account the phantom orbitals for optimized molecules in complexes and considering relaxation effects of isolated molecules. 

The distributions of electronic properties along the studied bonds were analyzed using electrostatic potential, υ_es_(**r**), static potential acting on an electron in a molecule, υ_st_(**r**), and electron delocalization indices δ(Tt|Hal) [68]. Since the υ_st_(**r**), in contrast to υ_es_(**r**), contains a contribution from exchange-correlation potential, υ_xc_(**r**), we estimate the latter as the difference between these quantities. For difference between extremes of potentials along the bond line, υ_st_(**r**)_max_ and υ_es_(**r**)_min_, we used υ_xc_(**r**)_ext_; for values at the critical point of electron density, the exchange-correlation potential was denoted as υ_xc_(**r**_cp_). We applied the evaluation of exchange-correlation density in terms of Müller approximation [69,70]. All these functions were calculated using Multiwfn program [71]. For calculating the Pauli potential, υ_P_(**r**), the approach suggested in [55] and implemented in Multiwfn was applied. Visualization of geometry and some properties of electron density at critical points, such as ρ(**r**_cp_), electrostatic potential, υ_es_(**r**_cp_), were calculated using AIMAll software package [72]. All calculated values for bonds properties of complexes Y–TtX_3_…Hal^−^ were gathered in Tables S2 and S3 of Supplementary Materials.

The quantitative relationships between E_bind_ and bond properties were analyzed using Statistica program [73].

## 3. Results and Discussion

### 3.1. Electron Density Properties

We analyzed the series of complexes Y–TtX_3_…Hal^−^ formed by halide anions Hal^−^ = F^−^, Cl^−^, Br^−^, and tetrahedral molecules Y–TtX_3_, in which the tetrel atom, Tt = C, Si, Ge, Sn, Pb, delivers its electrophilic region for bonding. The functional group Y = Hal, CH_3_, CN, NH_2_, NO_2_, covalently bound with the Tt atom was placed at the same line with it and the nucleophilic fragment Hal^−^. Each of the three identical substituents X = Cl, Br, CH_3_, was involved in interactions with Hal^–^. Note that the arrangement of three bulky electronegative substituents X_3_ on the side of a halide anion was influenced by the steric screening of the σ-hole for the Tt-atom. Nevertheless, this fact did not prevent the formation of the weak and strong Hal^−^…Tt bonds. We can point out the following features of the Y–TtX_3_…Hal^−^ complexes. Firstly, the geometry of the complexes essentially depends on the strength of the Hal^–^…Tt interaction. The long and weak Hal^−^…Tt bonds only slightly distort the tetrahedral shape of the Y–TtX_3_ molecule, while the short and strong bonds lead to the shape of trigonal bipyramid for a complex. The last effect is more often manifested for heavy Tt-atoms, but it also depends on the σ-hole polarizing introduced by the Y substituent. Secondly, we have found that not every weakly bound complex has a bond path and bond critical point (bcp) between the Tt-atom and Hal^−^. Thus, the combination of substituents in the Y–TtX_3_ molecule has made it possible to form the three types of Hal^−^…Tt interactions in our sample. There are the weakest interactions without a bond path between Hal^−^ and Tt, the typical tetrel bonds Hal^−^…Tt formed by the slightly distorted geometry of the tetrahedral molecule, and the strong bonds approaching the covalent character in the complexes which take the form of a trigonal bipyramid.

**Figure 1:**
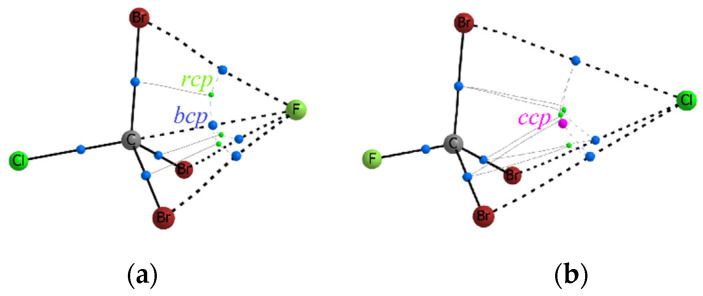
Different curvature of electron density and signature of cp in isoelectronic complexes (**a**) Cl–CCl_3_…F^−^ and (**b**) F–CCl_3_…Cl^–^.

For complexes in which Tt = C, it has been found that the bcp (3, −1) is formed only for the F^−^…C bonds. In other cases, we observe the arrangement of electron density curvature according to the cage type, with a cage critical point (ccp) (3, +3) approximately on the line Hal^−^…C, Hal = Cl^−^, Br^−^. In this case, any halide anion forms the bond paths with any substituent X = Cl, Br, CH_3_. It is interesting that in two isoelectronic complexes F–CCl_3_…Cl^−^ and Cl–CCl_3_…F^−^, the formation of completely different electron density curvature between the Hal^–^ and C atoms is observed (Figure 1). This observation confirms that the fluorine atom is not the best polarizing carbon substituent, but it is able to form the noncovalent bond F^−^…C with relatively high values of ρ(**r**_bcp_). The values of electron density at the cage critical points, ρ(**r**_ccp_), are almost an order of magnitude lower than ρ(**r**_bcp_) in complexes with a typical TtB.

**Figure 2:**
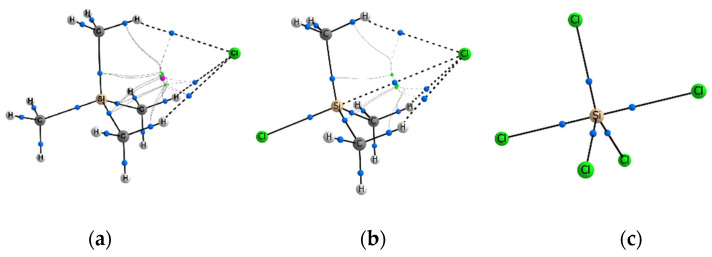
Different curvature of electron density and disposition of cp in complexes (**a**) CH_3_–Si(CH_3_)_3_…Cl^−^; (**b**) Cl–Si(CH_3_)_3_…Cl^−^; (**c**) Cl–SiCl_3_…Cl^−^.

In most cases where Tt = Si, we observe the bcp and bond path formation for the Hal^−^…Si interactions (Figure 2b), and it is one of the attributes of a typical tetrel bond (TtB). Nevertheless, the several Hal^−^…Si interactions are not strong enough. In the equilibrium state, the halide anion is relatively far from the Tt position, and the ccp is formed. Such a situation is observed for the cases of weakly polarizing substituents Y with an electron-donor property: Y = NH_2_, CH_3_ (Figure 2a). The participation of the F atom as a polarizing group does not contribute to the essential strengthening of the Hal^−^…Si interactions. In the F–Si(CH_3_)_3_…Cl^−^ and F–Si(CH_3_)_3_…Br^–^ complexes, the electron density in the region between the Si and Hal^–^ atoms is already higher than for Tt = C, but the Hal^–^…Si interatomic distance and the curvature of the electron density does not yet allow the bond path formation. Instead, we observe the formation of only two ring critical points (rcp) (3, +1) close to each other. In this case, C_3v_ symmetry is broken due to slightly different orientation of the methyl groups. Methyl groups in equatorial positions, X_3_ = CH_3_, do not create obstacles for the Hal^−^…Tt bond path; in addition, one hydrogen atom of each CH_3_ group forms the bound path with Hal^−^. In the complex with four identical substituents, Y = X_3_ = Cl, the resulting bond Cl^−^…Si is as strong as the one opposite of it (Figure 2c). If the F^−^ participates as a nucleophile, then the Hal^–^…Si bond path is formed, just as it was observed for complexes with Tt = C.

**Figure 3:**
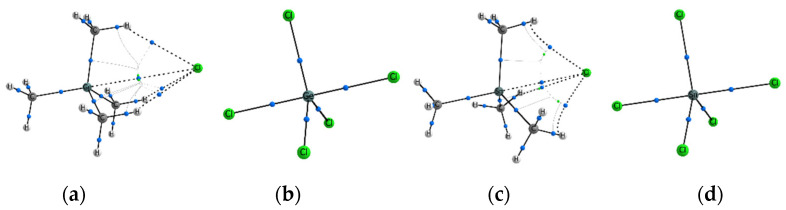
Weak and strong complexes (**a**) CH_3_–Ge(CH_3_)_3_…Cl^−^; (**b**) Cl–GeCl_3_…Cl^–^; (**c**) CH_3_–Sn(CH_3_)_3_…Cl^−^; (**d**) Cl–SnCl_3_…Cl^–^.

In all cases when Tt = Ge, only bond critical points of electron density are observed for Hal^−^…Ge interactions. Formation of bond paths confirms the presence of TtBs (Figure 3a). Nevertheless, another important feature begins to appear in a number of Y–GeX_3_…Hal^−^ complexes. The tetrahedral geometry of the Y–GeX_3_ molecule could be strongly distorted in complexes with relatively strong Hal^−^…Ge interactions. Due to the more pronounced ability of the Ge atom to be polarized, the Hal^−^ can come close to it, forming a rather short and strong Hal^−^…Ge bond. In such cases, the shape of the Y–GeX_3_ molecule is strongly distorted, three X substituents fit into one equatorial plane, and the geometry of the complex acquires the shape of a trigonal bipyramid. Relatively high values of ρ(**r**_bcp_) occur for Hal^−^…Ge bonds in such complexes (Table S2). They are comparable with ρ(**r**_bcp_) for Y–Ge covalent bonds located in axial positions, but they are slightly inferior to Ge–X covalent bonds in equatorial positions. Note that the bipyramid geometry of complexes is accompanied by the disappearance of all three Hal^−^…X bond paths, regardless of the substituents X = Cl, Br, CH_3_ (Figure 3b). Nevertheless, the effect of the X_3_ substituents can be illustrated by the following example. For the F–GeX_3_…Cl^−^ complexes, the binding energy, E_bind_, decreases in the order X_3_ = F < Br < Cl, while ρ(**r**_bcp_) decreases in the different order X_3_ = Br < F < Cl. This fact indicates the steric effect created by three atoms X = Br, which restrain a shorter Cl^−^…Ge bond formation.

For most complexes Y–TtX_3_…Hal^–^, Tt = Sn, Pb, we observe a relatively strong formation of Hal^−^…Tt bonds formation (Figure 3b,d) accompanied by bcp with rather high values of ρ(**r**_bcp_). The geometry of the complexes takes the form of a trigonal bipyramid, as a rule. Nevertheless, in the series of Tt = Sn complexes, there are still cases where the tetrahedral form of the Y–Sn(CH_3_)_3_ molecules is retained, where the electron-donor groups NH_2_ and CH_3_ participate as Y substituent (Figure 3a,c). It can be concluded that the effect created by the polarizing group Y is important, and its manifestation is clearly visible in the series from Si to Sn. For Tt = Pb, all complexes are trigonal bipyramids and they are characterized by a low negative binding energy, E_bind_, and ρ(**r**_bcp_) between Hal^–^ and Pb is equal or comparable to the values for the covalent bonds of the corresponding sort.

The fact that a number of studied bonds formed by the Tt atom approaches the bonds with covalent characters can be supported by a comparison of the properties of the axial Hal^−^…Tt and equatorial Tt–X bonds of the same sort for which Hal^−^ = X, Tt = Si, Ge, Sn, Pb (Table S1). The positions of maxima for the ρ(**r**_bcp_) normal distributions are quite close to each other and amount to 0.069 and 0.076 a.u., respectively (Figure S1). The total range of the ρ(**r**_bcp_) values is from 0.04 to 0.14 a.u. A comparison of the axial bonds of one complex Hal^–^…Tt and Y–Tt for which Hal = Y leads to the normal distributions of ρ(**r**_bcp_) with maxima at 0.074 and 0.080 a.u., respectively (Figure S2a). If we take into account the normal distributions of the weak Hal^–^…C bonds, formed by Y–CX_3_ molecules with a small distortion of the tetrahedral shape, then the difference in the maxima positions will amount to 0.05 a.u. (Figure S1b).

Thus, for the series of the Y–TtX_3_…Hal^–^ complexes considered in our study, the strength of the Hal^–^…Tt interactions increases with an increase in the atomic number of Tt in the series from C to Pb. Note that the widest range of the type of Hal^–^…Tt interactions falls on the case of Tt = Si. At the same time, we should take into account that TtBs in our set of complexes are under the influence of a number of factors created by different substituents. For example, in a number of cases, the nucleophilic Hal^–^, in addition to the TtB formation with an electrophilic region of the Tt atom, can be involved in noncovalent interactions with electronegative substituents, X_3_.

### 3.2. Electronic Criterion for Weak and Strong Bonds Involving Tt

When a covalent bond is formed, a variational balance is achieved between the kinetic and potential energy of electrons. Let us consider a potential component and turn to the electronic criterion [23] based on the electron density and electrostatic potential distributions. It has been created for identifying the electrophilic region and for naming the noncovalent bonds in cases of “non-obvious σ-hole—lone pair” mutual orientation. In accordance with the electronic criterion, the minimum of electrostatic potential along the bond line is closer to the atom that shows the local accumulation of electron density. The minimum of electron density is always located closer to the atom that provides its electrophilic site for bonding.

**Figure 4:**
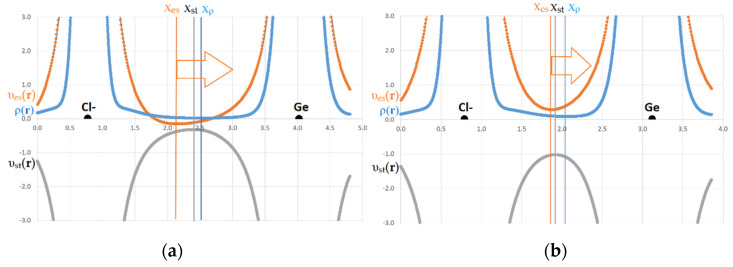
The disposition of electron density, and electrostatic potential minima and potential acting on an electron in a molecule, (a.u.) along interatomic line Hal^–^…Ge in complexes (**a**) F–Ge(CH_3_)_3_…Cl^−^ and (**b**) F–GeF_3_… Cl^−^. The arrows point to the atom providing electrophilic region. Vertical lines show the extremes of functions; corresponding color is used.

The application of the electronic criterion to the considered complexes with weak and strong interactions of the Tt atom illustrates the following results. The behavior of the electrostatic potential, υ_es_(**r**), static potential, or potential acting on an electron in a molecule, υ_st_(**r**), electron density functions, ρ(**r**), has an extreme character along the line of the Hal^–^…Tt bond (Figure 4). A comparison of the extreme positions x_es_, x_st_, x_ρ_, of the abovementioned functions (Table S2) shows that for all considered cases, the minimum of the ρ(**r**) is located on the side of the Tt atom, which provides its electrophilic region for bonding, and the minimum of υ_es_(**r**) is closer to nucleophilic Hal^–^. Position x_ρ_ corresponds to the boundary of Bader’s atomic basins Hal^–^|Tt, and position x_es_ separates the electrically neutral atomic basins of these atoms. The observed ranking of the minima positions, x_st_|x_ρ_, mirrors the superposition of the boundaries of neutral and charged atomic basins. For all Hal^–^…Tt interactions, it has been confirmed that the Tt atom provides its electrophilic region for bonding and the fraction of electrons belonging to Hal^–^ is attracted to the nucleus of the Tt atom, as it should be for a typical TtB. Note that, for shorter and stronger Hal^–^…Tt bonds, the gap between x_st_ and x_ρ_ is significantly smaller than for longer and weaker bonds. This fact confirms the observations presented in [74].

It is important that the x_st_ coordinate which determines the position of the static potential maximum, υ_st_(**r**)_max_, along the bond line, is always located between the x_es_ and x_ρ_ positions. We denote the gap between the positions of υ_st_(**r**)_max_ and υ_es_(**r**)_min_ as (x_st_ – x_es_) = ∆_st-es_. The gap between the υ_st_(**r**)_max_ and ρ(**r**)_min_ positions is labeled as (x_ρ_ – x_st_) = ∆_ρ__-st_. By analyzing the positions x_es_, x_st_, and x_ρ_ in the series of Hal^–^…Tt bonds, we have discovered the following phenomenon. The gaps ∆_st-es_ and ∆_ρ__-st_ differ significantly for weak and strong Hal^–^…Tt bonds and depend on the sort of Hal^–^ and Tt atoms. If we count from the position of the Hal^–^, then for relatively weak noncovalent bonds of Hal^–^…Tt, ∆_st-es_ > ∆_ρ__-st_ (or Δx = ∆_st-es_ – ∆_ρ__-st_ > 0). It means that the gap between the positions of υ_st_(**r**)_max_ and υ_es_(**r**)_min_ is significantly wider than the gap between the positions of υ_st_(**r**)_max_ and ρ(**r**)_min_. For relatively strong bonds that have acquired a partially covalent character, everything is exactly the opposite: ∆_st-es_ < ∆_ρ__-st_ (or Δx = ∆_st-es_ – ∆_ρ__-st_ < 0).

**Figure 5:**
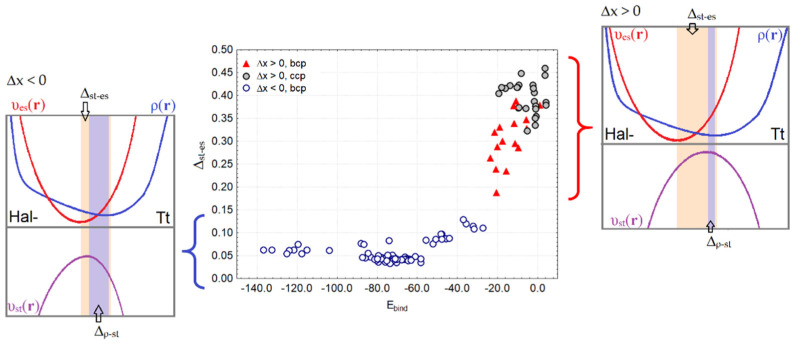
The gap width between positions of electrostatic potential minimum and static potential maximum, (Å) along interatomic line Hal^−^…Tt related with the binding energy in Y–TtX_3_…Hal^−^ complexes.

We tested the width of the two gaps ∆_st-es_ and ∆_ρ__-st_ for their correlations with the Hal^–^…Tt bond lengths, binding energy E_bind_, and electron density properties characterizing the features of weak and strong bonds in the complexes under study. As Figure 5 shows, the relationship between the gap width ∆_st-es_ and E_bind_ follows a certain logic. For relatively weak bonds of Hal^–^…Tt, for which Δx > 0, regardless of the curvature of the electron density and the signature of its critical points between Hal^–^ and Tt, the binding energy E_bind_ does not exceed –24 kcal/mol. The gap width ∆_st-es_ for such noncovalent bonds varies significantly in a wide range from 0.17 to 0.47 Å. Note that for interactions between Hal^–^ and Tt that are not accompanied by bcp and a bond path, the gap ∆_st-es_ is only slightly larger than for typical TtBs characterized by bcp presence. For relatively strong Hal^–^…Tt bonds with E_bind_ in the range from –24 to –140 kcal/mol, the gap ∆_st-es_ is small and changes in the narrow range from 0.05 to 0.10 Å (Figure 5). Note that noncovalent bonds involving Tt = C do not fall into the range of strong bonds, just as the bonds Tt = Pb do not fall into the range of weak ones. The width of another gap ∆_ρ__-st_ remains dependent on the bond sort; nevertheless, it does not perfectly correlate with the binding energy or electron density properties characterizing the strength of Tt interactions in complexes.

**Figure 6:**
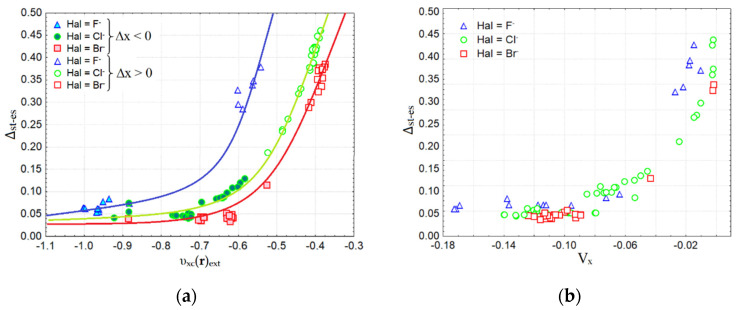
Relationships between the gap ∆_st-es_ along interatomic line Hal^−^…Tt and the exchange-correlation contribution υ_xc_(**r**)_ext_ in the static potential at extrema of υ_es_(**r**)_min_ and υ_st_(**r**)_max_ (**a**) and with the bond contribution to exchange energy, V_x_ (**b**).

Next, we focused our attention on the quantitative relationships between the gap width ∆_st-es_ and the energy characteristics of Hal^–^…Tt bonds. It has been found that the value ∆_st-es_ depends exponentially on the exchange-correlation contribution, υ_xc_(**r**)_ext_, to the static potential, υ_st_(**r**) (Figure 6a). For each sort of Hal^–^…Tt bond determined by the nucleophilic fragment Hal^–^ = F^–^, Cl^–^, Br^–^, a separate exponential dependence is observed.

∆_st-es_ = a_0_ ⋅ exp[a_1_ ⋅ υ_xc_(**r**)] (7)

The fitting parameters in Equation (7) for the separate sets with various nucleophilic fragments are given in Table 1. For weak Hal^–^…Tt bonds with relatively small values of υ_xc_(**r**)_ext_ (–0.35 ÷ –0.65 a.u.), the gap width ∆_st-es_ rapidly decreases with increasing the bond length in the range from 0.15 to 0.50 Å. At the same time, a similar rate of exponential fall is observed for different sorts of TtBs determined by Hal^–^. Note that for Hal = F^–^, the values of υ_xc_(**r**)_ext_ are much larger in their absolute value than for Hal = Cl^–^, Br^–^. For strong bonds of any sort, the great negative values υ_xc_(**r**)_ext_ = –0.50 ÷ –1.05 a.u. correspond to the small common range (0.01 ÷ 0.15 Å) of the gap between the positions of the electrostatic potential minimum, υ_es_(**r**)_min_, and static potential maximum, υ_st_(**r**)_max_, along the Hal^–^…Tt bond line.

Thus, it can be stated that strong and weak bonds of any sort are unambiguously separated in the scale of the gap width ∆_st-es_ in the region of 0.15 ± 0.02 Å. For strong Hal^–^…Tt bonds in our series, the type of which can be defined as a covalent, the gap width (x_st_ – x_es_) reaches a certain limit; for weak noncovalent bonds, the influence of the sort of Hal^–^ is clearly manifested. The observation that, for weak noncovalent bonds, the υ_xc_(**r**)_ext_ contribution to the static potential is strongly tied to Hal^–^ properties, can be explained by the fact that at long distances, the individuality of bound atoms and specific features of electron density distribution for nucleophilic fragments are preserved.

We have also made sure that the exponential nature of the ∆_st-es_ vs. υ_xc_(**r**) dependence is preserved if we evaluate the exchange-correlation contribution to the static potential at the critical points of electron density (in this case, the notation υ_xc_(**r**_bcp_) is used). Considering the largest set of bonds Cl^–^…Tt, we have verified that for weak interactions, υ_xc_(**r**_bcp_) is sensitive to X_3_ substituents, and depends on Y atom variation, which polarizes the Tt atom that mainly leads to the enhancement of the TtB strength. In addition, we note that in conditions of fixed nucleophilic fragments, the exponential trend [∆_st-es_ vs. υ_xc_(**r**)] remains common for various Tt atoms (Tt = Si, Ge, Sn, Pb).

We could estimate the contribution to the electron exchange energy from Hal^–^…Tt bonds by the expression V_x_ = −δ(Hal|Tt)/2R(Hal…Tt) [75,76], where δ(Hal|Tt) is the electron delocalization indices and R(Hal…Tt) is the interatomic distance. It can be seen that each nucleophilic fragment, F^–^, Cl^–^, and Br^–^, is responsible for its own trend, which is not exponential (Figure 6b). For very weak interactions of any sort, V_x_ asymptotically tends to zero. The narrowest gap ∆_st-es_ ~ 0.05 Å corresponds to values V_x_ < −0.10 a.u.

Most properties of the chemical bonds are strongly dependent on the sort of bound atoms. The accumulated observations of the quantum-topological characteristics of electron density [77] rather indicate the absence of a specific boundary between covalent and noncovalent bonds, so it would be reasonable to speak about the intermediate type of bonds. Nevertheless, the possibility of the separation of the interactions of our set into weak and strong bonds suggests determining a certain “reference point”, for example, on the scale of energy or properties of electron density that could mark the threshold for covalent and noncovalent bonds. Such a step could be important for the express determination of a chemical bond type.

**Figure 7:**
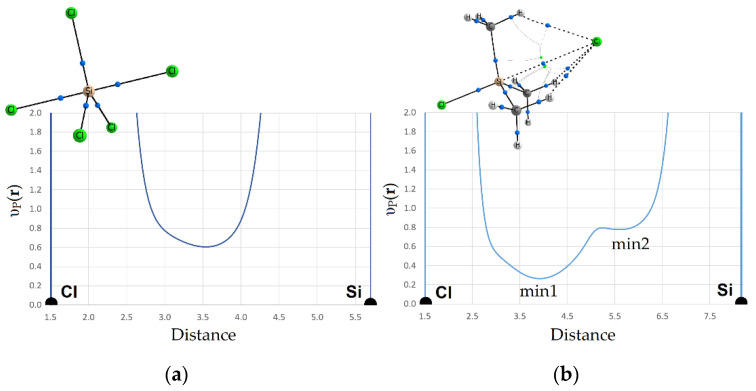

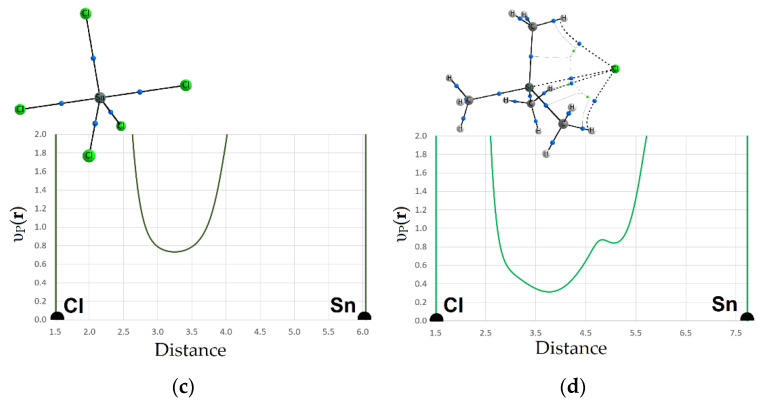
The distribution of Pauli potential (a.u.) along interatomic line Hal^–^…Tt in complexes (**a**) Cl–SiCl_3_…Cl^–^, (**b**) Cl–Si(CH_3_)_3_…Cl^–^, (**c**) Cl–SnCl_3_…Cl^–^, and (**d**) Cl–Sn(CH_3_)_3_…Cl^–^.

It is clear that the switching of the bond type from covalent to noncovalent demands the transfer from the sharing of an electron pair by two atoms forming a strong bond to another bonding mechanism. The kinetic component of bonding can help to better understand the resulting picture. The Pauli potential, υ_P_(**r**), reflects important kinetic features of electron pairing and can serve as the characteristic that allows us to find out the threshold between covalent and noncovalent bonds in our series. Let us compare the behavior of the υ_P_(**r**) function along the bond line for strong and weak interactions of the same sort. As it turned out, the Pauli potential, υ_P_(**r**), exhibits two (sometimes more) minima in the central part of the weak noncovalent bonds (Figure 7). Each of these minima characterizes the positions in which the ability to form a pair of electrons is slightly higher in comparison to the local maxima or barriers. Note that the minimum from the side of Hal^–^, υ_P_(**r**)_min1_, is always deeper than the next neighboring minimum υ_P_(**r**)_min2_ located at the side of the Tt atom, providing its local electrophilic region for bonding. While the enhanced local positive electrostatic potential favors the electron transfer to the electrophilic region of the Tt atom, the Pauli potential, υ_P_(**r**), indicates the unfavorable regions for the entry of electrons into this region controlling the electron pair formation.

For weak Hal^–^…Tt bonds, the minima υ_P_(**r**)_min1_ and υ_P_(**r**)_min2_ are separated by a small barrier which apparently indicates that a common region for an sharing electron pair cannot yet be formed, and the electronic features of Hal^–^ and Tt atoms still retain their originality. An important finding was that two or more minima of Pauli potential, υ_P_(**r**), which were formed for weak Hal^–^…Tt interactions satisfied the condition (x_st_ – x_es_) > (x_ρ_ – x_st_) or Δx > 0. At the same time, the positions of the electrostatic and total static potentials extremes, υ_es_(**r**)_min_ and υ_st_(**r**)_min_, could not coincide with the υ_P_(**r**)_min1_ or υ_P_(**r**)_min2_ positions. In only two cases for the H_2_N–Sn(CH_3_)_3_…Cl^–^ and F–Ge(CH_3_)_3_…Cl^–^ complexes, for which Δx > 0, we observed the absence of the second minimum υ_st_(**r**)_min2_.

In all cases of strong bonds of Hal^–^…Tt, for which the condition ∆_st-es_ < ∆_ρ-st_ or Δx < 0 is satisfied, the Pauli potential forms only one minimum between the Hal^–^ and Tt atoms. This fact can be interpreted as the presence of a common region in which electron pair formation is allowed; such a case indicates the covalent nature of the bond.

Thus, our finding that the gap widths between positions of extremes for the static potential, υ_st_(**r**), and electrostatic potential, υ_es_(**r**), is in accordance with the behavior of the Pauli potential, υ_P_(**r**), along the bond line, gives evidence that the threshold demarcation of the covalent and noncovalent bonds in the series of Hal^–^…Tt interactions has a physical basis.

## 4. Conclusions

A need to determine the range of properties for typical TtB bonds prompted us to analyze the electronic properties in a series of Y–TtX_3_…Hal^–^ complexes with a gradual increase of Hal^–^…Tt bonds’ strength under the influence of various substituents. As a rule, the energy characteristics and quantum-topological features of electron density rather indicate the absence of a clear threshold between not very strong and not so weak chemical bonds. It might seem that in a series illustrating the smooth strengthening of bonds under the influence of various substituents it makes sense to talk about interactions of intermediate nature. Nevertheless, the summary of our observations of the electrostatic potential, υ_es_(**r**), static potential, υ_st_(**r**), and Pauli potential, υ_P_(**r**), distributions between the Hal^–^ and Tt atoms do not support this supposition.

One or two minima of the Pauli potential, υ_P_(**r**), along the bond line Hal^–^…Tt suggest that the balance of forces and the features of electron distribution between the Hal^–^ and Tt atoms is different for strong bonds, approaching the covalent type, and for weak noncovalent bonds. One minimum of υ_P_(**r**) favors the formation of a common electron pair, while two minima of υ_P_(**r**) indicate the preservation of the specificity of atomic shells in a typical noncovalent bond. In addition, we did not observe noticeable differences in the behavior of the Pauli potential, υ_P_(**r**), for weak interactions with a bond critical point and cage critical point between the atoms.

The gap ∆_st-es_ between positions of extremes for electrostatic, υ_es_(**r**), and static, υ_st_(**r**), potentials along the Hal^–^…Tt line is wide for weak bonds and narrow for strong ones. It is in very good agreement with the number of minima of the Pauli potential, υ_P_(**r**), at the middle of a bond. This observation allowed us to propose a criterion based on the comparison of the gap widths ∆_st-es_ and ∆_ρ-st_, which can serve as an informative descriptor for the express establishment of the type of bond. For covalent bonds, ∆_st-es_ < ∆_ρ-st_, whereas, on the contrary, for noncovalent TtB bonds, ∆_st-es_ > ∆_ρ-st_. The gap width ∆_st-es_ exponentially correlates with the exchange-correlation component of static potential in a series of the bonds Hal^–^…Tt with the fixed nucleophilic fragment Hal^–^.

Thus, the analysis of the electrostatic, static, and Pauli potentials along the line between the Hal^–^ and Tt atoms has made it possible to verify that a priori attribution of interactions to covalent or noncovalent bonds and their categorization according to the electrophilic region has all the physical grounds supported by quantitative data.

A preliminary check confirms the transferability of the suggested criteria to the series of complexes between the neutral molecules with strong and weak tetrel bonds. The results will be reported later.

## Figures and Tables

**Table 1:** Parameters of Equation (7) describing relationship between the gap ∆_st-es_ (Å) along interatomic line Hal^–^…Tt and the contribution of exchange-correlation potential υ_xc_(**r**), (a.u.) estimated on the base of extremes of potentials and at the critical points of electron density (in brackets).

Type of the Set, (Number of Cases)	a_0_	a_1_	R, Correlation Coefficient
Hal = F^–^, (19)	3.515 (3.913)	4.144 (5.138)	0.99 (0.99)
Hal = Cl^–^, (56)	5.550 (3.416)	6.468 (6.279)	0.97 (0.96)
Hal = Br^–^, (36)	6.600 (4.945)	7.705 (7.824)	0.97 (0.98)

## Data Availability

All data are available within the article or Supplementary Materials.

## Supplementary Materials

The following supporting information can be downloaded at: https://www.mdpi.com/article/10.3390/molecules27175411/s1, Figure S1: Histograms of electron density distribution at bcp for axial Hal^−^…Tt and equatorial Tt–X bonds of the same type, Hal^−^ = X, in series of complexes Y–TtHal_3_…Hal^–^, Tt = Si, Ge, Sn, Pb; Figure S2: Figure S2: Histogram of electron density distribution at bcp between axial Hal^−^…Tt and Y–Tt bonds of the same type, Hal^–^ = Y, in series of complexes Y–TtHal_3_… Hal^–^ (a) Tt = Si, Ge, Sn, Pb; (b) Tt = C, Si, Ge, Sn, Pb; Table S1: Bond lengths and electron density at critical points (a. u.) for Tt–Y and Tt–Hal interactions in complexes Y–TtX_3_… Hal^–^; Table S2: Properties of the static, electrostatic, and Pauli potentials for Hal^–^…Tt bonds in complexes Y–TtX_3_… Hal^–^; Table S3: The static and electrostatic potentials, exchange-correlation contribution in total static potential at the critical points of electron density, and bond contribution to exchange energy for Hal^–^…Tt bonds in complexes Y–TtX_3_… Hal^–^.

## Sample Availability

Samples of the compounds are available from the authors.

## References

[1] Legon A.C. (2017). Tetrel, Pnictogen and Chalcogen Bonds Identified in the Gas Phase before They Had Names: A Systematic Look at Non-Covalent Interactions. Phys. Chem. Chem. Phys..

[2] Terraneo G., Resnati G. (2017). Bonding Matters. Cryst. Growth Des..

[3] Politzer P., Murray J.S. (2019). A Look at Bonds and Bonding. Struct. Chem..

[4] Politzer P., Murray J.S. (2002). The Fundamental Nature and Role of the Electrostatic Potential in Atoms and Molecules. Theor. Chem. Acc..

[5] Politzer P., Murray J.S., Clark T. (2010). Halogen Bonding: An Electrostatically-Driven Highly Directional Noncovalent Interaction. Phys. Chem. Chem. Phys..

[6] Desiraju G.R., Ho P.S., Kloo L., Legon A.C., Marquardt R., Metrangolo P., Politzer P., Resnati G., Rissanen K. (2013). Definition of the Halogen Bond (IUPAC Recommendations 2013). Pure Appl. Chem..

[7] Aakeroy C.B., Bryce D.L., Desiraju G.R., Frontera A., Legon A.C., Nicotra F., Rissanen K., Scheiner S., Terraneo G., Metrangolo P. (2019). Definition of the Chalcogen Bond (IUPAC Recommendations 2019). Pure Appl. Chem..

[8] Alkorta I., Elguero J., Frontera A. (2020). Not Only Hydrogen Bonds: Other Noncovalent Interactions. Crystals.

[9] Grabowski S.J. (2014). Tetrel Bond–σ-Hole Bond as a Preliminary Stage of the SN2 Reaction. Phys. Chem. Chem. Phys..

[10] Sethio D., Oliveira V., Kraka E. (2018). Quantitative Assessment of Tetrel Bonding Utilizing Vibrational Spectroscopy. Molecules.

[11] Lu J., Scheiner S. (2019). Effects of Halogen, Chalcogen, Pnicogen, and Tetrel Bonds on IR and NMR Spectra. Molecules.

[12] Hou M., Liu Z., Li Q. (2020). The π-Hole Tetrel Bond between X2TO and CO_2_: Substituent Effects and Its Potential Adsorptivity for CO_2_. Int. J. Quantum Chem..

[13] Scheiner S. (2018). Steric Crowding in Tetrel Bonds. J. Phys. Chem. A.

[14] Scheiner S. (2021). Origins and Properties of the Tetrel Bond. Phys. Chem. Chem. Phys..

[15] Zierkiewicz W., Michalczyk M., Scheiner S. (2018). Comparison between Tetrel Bonded Complexes Stabilized by σ and π Hole Interactions. Molecules.

[16] Grabowski S.J., Scheiner S. (2018). Tetrel Bonds with π-Electrons Acting as Lewis Bases—Theoretical Results and Experimental Evidences. Molecules.

[17] Scheiner S. (2018). Tetrel Bonding as a Vehicle for Strong and Selective Anion Binding. Molecules.

[18] Liu M., Li Q., Cheng J., Li W., Li H.B. (2016). Tetrel Bond of Pseudohalide Anions with XH3F (X = C, Si, Ge, and Sn) and Its Role in SN2 Reaction. J. Chem. Phys..

[19] Frontera A., Bauzá A. (2018). S⋅⋅⋅Sn Tetrel Bonds in the Activation of Peroxisome Proliferator-Activated Receptors (PPARs) by Organotin Molecules. Chem. A Eur. J..

[20] Daolio A., Scilabra P., Terraneo G., Resnati G. (2020). C(Sp3) Atoms as Tetrel Bond Donors: A Crystallographic Survey. Coord. Chem. Rev..

[21] Scilabra P., Kumar V., Ursini M., Resnati G. (2018). Close Contacts Involving Germanium and Tin in Crystal Structures: Experimental Evidence of Tetrel Bonds. J. Mol. Model..

[22] Bartashevich E., Matveychuk Y., Tsirelson V. (2019). Identification of the Tetrel Bonds between Halide Anions and Carbon Atom of Methyl Groups Using Electronic Criterion. Molecules.

[23] Bartashevich E., Mukhitdinova S., Yushina I., Tsirelson V. (2019). Electronic Criterion for Categorizing the Chalcogen and Halogen Bonds: Sulfur-Iodine Interactions in Crystals. Acta Crystallogr. Sect. B Struct. Sci. Cryst. Eng. Mater..

[24] Bauzá A., Seth S.K., Frontera A. (2019). Tetrel Bonding Interactions at Work: Impact on Tin and Lead Coordination Compounds. Coord. Chem. Rev..

[25] Kumar V., Rodrigue C., Bryce D.L. (2020). Short and Linear Intermolecular Tetrel Bonds to Tin. Cocrystal Engineering with Triphenyltin Chloride. Cryst. Growth Des..

[26] Bader R.F.W. (1990). Atoms in Molecules. A Quantum Theory.

[27] Bader R.F.W., Essén H. (1998). The Characterization of Atomic Interactions. J. Chem. Phys..

[28] Cremer D., Kraka E. (1984). A Description of the Chemical Bond in Terms of Local Properties of Electron Density and Energy. Croat. Chem. Acta.

[29] Espinosa E., Molins E., Lecomte C. (1998). Hydrogen Bond Strengths Revealed by Topological Analyses of Experimentally Observed Electron Densities. Chem. Phys. Lett..

[30] Mata I., Alkorta I., Espinosa E., Molins E. (2011). Relationships between Interaction Energy, Intermolecular Distance and Electron Density Properties in Hydrogen Bonded Complexes under External Electric Fields. Chem. Phys. Lett..

[31] Espinosa E., Alkorta I., Elguero J., Molins E. (2002). From Weak to Strong Interactions: A Comprehensive Analysis of the Topological and Energetic Properties of the Electron Density Distribution Involving X–H⋯F–Y Systems. J. Chem. Phys..

[32] Vener M.V., Egorova A.N., Churakov A.V., Tsirelson V.G. (2012). Intermolecular Hydrogen Bond Energies in Crystals Evaluated Using Electron Density Properties: DFT Computations with Periodic Boundary Conditions. J. Comput. Chem..

[33] Bushmarinov I.S., Lyssenko K.A., Antipin M.Y. (2009). Atomic Energy in the “Atoms in Molecules” Theory and Its Use for Solving Chemical Problems. Russ. Chem. Rev..

[34] Ananyev I.V., Karnoukhova V.A., Dmitrienko A.O., Lyssenko K.A. (2017). Toward a Rigorous Definition of a Strength of Any Interaction between Bader’s Atomic Basins. J. Phys. Chem. A.

[35] Bartashevich E.V., Tsirelson V.G. (2014). Interplay between Non-Covalent Interactions in Complexes and Crystals with Halogen Bonds. Russ. Chem. Rev..

[36] Kuznetsov M.L. (2019). Can Halogen Bond Energy Be Reliably Estimated from Electron Density Properties at Bond Critical Point? The Case of the (A)NZ—Y•••X− (X, Y  =  F, Cl, Br) Interactions. Int. J. Quantum Chem..

[37] Kuznetsov M.L., Costa P.J. (2019). Relationships between Interaction Energy and Electron Density Properties for Homo Halogen Bonds of the [(A)NY–X···X–Z(B)m] Type (X = Cl, Br, I). Molecules.

[38] Bartashevich E.V., Tsirelson V.G. (2013). Atomic Dipole Polarization in Charge-Transfer Complexes with Halogen Bonding. Phys. Chem. Chem. Phys..

[39] Alkorta I., Legon A.C. (2017). Nucleophilicities of Lewis Bases b and Electrophilicities of Lewis Acids a Determined from the Dissociation Energies of Complexes B··· A Involving Hydrogen Bonds, Tetrel Bonds, Pnictogen Bonds, Chalcogen Bonds and Halogen Bonds. Molecules.

[40] Bartashevich E.V., Matveychuk Y.V., Mukhitdinova S.E., Sobalev S.A., Khrenova M.G., Tsirelson V.G. (2020). The Common Trends for the Halogen, Chalcogen, and Pnictogen Bonds via Sorting Principles and Local Bonding Properties. Theor. Chem. Acc..

[41] Wesolowski T.A., Wang Y.A. (2013). Recent Progress in Orbital-Free Density Functional Theory.

[42] Tsirelson V., Stash A. (2020). Orbital-Free Quantum Crystallography: View on Forces in Crystals. Acta Crystallogr. Sect. B Struct. Sci. Cryst. Eng. Mater..

[43] Tsirelson V., Stash A. (2021). Developing Orbital-Free Quantum Crystallography: The Local Potentials and Associated Partial Charge Densities. Acta Crystallogr. Sect. B Struct. Sci. Cryst. Eng. Mater..

[44] Zhao D.X., Gong L.D., Yang Z.Z. (2005). The Relations of Bond Length and Force Constant with the Potential Acting on an Electron in a Molecule. J. Phys. Chem. A.

[45] Zhao D.-X., Yang Z.-Z. (2014). Investigation of the Distinction between van Der Waals Interaction and Chemical Bonding Based on the PAEM-MO Diagram. J. Comput. Chem..

[46] Bartashevich E., Tsirelson V. (2018). A Comparative View on the Potential Acting on an Electron in a Molecule and the Electrostatic Potential through the Typical Halogen Bonds. J. Comput. Chem..

[47] Hamilton I.P., Mosna R.A., Site L.D. (2007). Classical Kinetic Energy, Quantum Fluctuation Terms and Kinetic-Energy Functionals. Theor. Chem. Acc..

[48] Liu S. (2007). Steric Effect: A Quantitative Description from Density Functional Theory. J. Chem. Phys..

[49] Delle Site L. (2002). Bader’s Interatomic Surface and Bohmian Mechanics. Europhys. Lett..

[50] March N.H. (2010). Concept of the Pauli Potential in Density Functional Theory. J. Mol. Struct. THEOCHEM.

[51] Shteingolts S.A., Stash A.I., Tsirelson V.G., Fayzullin R.R. (2021). Orbital-Free Quantum Crystallographic View on Noncovalent Bonding: Insights into Hydrogen Bonds, Π⋅⋅⋅π and Reverse Electron Lone Pair⋅⋅⋅π Interactions. Chem. Eur. J..

[52] Levina E.O., Khrenova M.G., Tsirelson V.G. (2021). The Explicit Role of Electron Exchange in the Hydrogen Bonded Molecular Complexes. J. Comput. Chem..

[53] Stash A.I., Terekhova E.O., Ivanov S.A., Tsirelson V.G. (2021). X-ray Diffraction Study of the Atomic Interactions, Anharmonic Displacements and Inner-Crystal Field in Orthorhombic KNbO3. Acta Crystallogr. Sect. B Struct. Sci. Cryst. Eng. Mater..

[54] Bartashevich E., Stash A., Yushina I., Minyaev M., Bolshakov O., Rakitin O., Tsirelson V. (2021). Bonding Features in Appel’s Salt from the Orbital-Free Quantum Crystallographic Perspective. Acta Crystallogr. Sect. B Struct. Sci. Cryst. Eng. Mater..

[55] Tsirelson V.G., Stash A.I., Karasiev V.V., Liu S. (2013). Pauli Potential and Pauli Charge from Experimental Electron Density. Comput. Theor. Chem..

[56] Astakhov A.A., Stash A.I., Tsirelson V.G. (2016). Improving Approximate Determination of the Noninteracting Electronic Kinetic Energy Density from Electron Density. Int. J. Quantum Chem..

[57] Gritsenko O.V., Mentel M., Baerends E.J. (2016). On the Errors of Local Density (LDA) and Generalized Gradient (GGA) Approximations to the Kohn-Sham Potential and Orbital Energies. J. Chem. Phys..

[58] Herring C. (1986). Explicit Estimation of Ground-State Kinetic Energies from Electron Densities. Phys. Rev. A.

[59] Bartashevich E., Yushina I., Kropotina K., Muhitdinova S., Tsirelson V. (2017). Testing the Tools for Revealing and Characterizing the Iodine-Iodine Halogen Bond in Crystals. Acta Crystallogr. Sect. B Struct. Sci. Cryst. Eng. Mater..

[60] Bartashevich E.V., Yushina I.D., Stash A.I., Tsirelson V.G. (2014). Halogen Bonding and Other Iodine Interactions in Crystals of Dihydrothiazolo(Oxazino)Quinolinium Oligoiodides from the Electron-Density Viewpoint. Cryst. Growth Des..

[61] Granovsky A.A. Firefly, Version 8. http://classic.chem.msu.su/gran/firefly/index.html.

[62] Schmidt M.W., Baldridge K.K., Boatz J.A., Elbert S.T., Gordon M.S., Jensen J.H., Koseki S., Matsunaga N., Nguyen K.A., Su S. (1993). General Atomic and Molecular Electronic Structure System. J. Comput. Chem..

[63] Adamo C., Barone V. (1999). Toward Reliable Density Functional Methods without Adjustable Parameters: The PBE0 Model. J. Chem. Phys..

[64] Barbieri P.L., Fantin P.A., Jorge F.E. (2006). Gaussian Basis Sets of Triple and Quadruple Zeta Valence Quality for Correlated Wave Functions. Mol. Phys..

[65] MacHado S.F., Camiletti G.G., Neto A.C., Jorge F.E., Jorge R.S. (2009). Gaussian Basis Set of Triple Zeta Valence Quality for the Atoms from K to Kr: Application in DFT and CCSD(T) Calculations of Molecular Properties. Mol. Phys..

[66] Campos C.T., Jorge F.E. (2012). Triple Zeta Quality Basis Sets for Atoms Rb through Xe: Application in CCSD(T) Atomic and Molecular Property Calculations. Mol. Phys..

[67] Pritchard B.P., Altarawy D., Didier B., Gibson T.D., Windus T.L. (2019). New Basis Set Exchange: An Open, Up-to-Date Resource for the Molecular Sciences Community. J. Chem. Inf. Model..

[68] Fradera X., Austen M.A., Bader R.F.W. (1999). The Lewis Model and Beyond. J. Phys. Chem. A.

[69] Müller A.M.K. (1984). Explicit Approximate Relation between Reduced Two- and One-Particle Density Matrices. Phys. Lett. A.

[70] Buijse M.A., Baerends E.J. (2009). An Approximate Exchange-Correlation Hole Density as a Functional of the Natural Orbitals. Mol. Phys..

[71] Lu T., Chen F. (2012). Multiwfn: A Multifunctional Wavefunction Analyzer. J. Comput. Chem..

[72] Keith T.A. AIMAll (Version 17.11.14). http://www.aim.tkgristmill.com.

[73] TIBCO Statistica, v. 13. https://www.tibco.com/products/tibco-statistica.

[74] Chakalov E.R., Tupikina E.Y., Bartashevich E.V., Ivanov D.M., Tolstoy P.M. (2022). The Distance between Minima of Electron Density and Electrostatic Potential as a Measure of Halogen Bond Strength. Molecules.

[75] Mayer I. (2014). Covalent Bonding: The Role of Exchange Effects. J. Phys. Chem. A.

[76] Outeiral C., Vincent M.A., Martín Pendás Á., Popelier P.L.A. (2018). Revitalizing the Concept of Bond Order through Delocalization Measures in Real Space. Chem. Sci..

[77] Tsirelson V.G. (2014). Quantum Chemistry. Molecules, Molecular Systems and Solids.

