# Supplementary material for: Can We Merge the Weak and Strong Tetrel Bonds? Electronic Features of Tetrahedral Molecules Interacted with Halide Anions
Source: Molecules. 2022 Aug 24;27(17):5411. doi: 10.3390/molecules27175411 (PMC9458139; doi:10.3390/molecules27175411)
Supplement: Supplementary file 1 [file molecules-27-05411-s001.zip › molecules-1849439-supplementary.pdf]

Supplementary Materials

Can we merge the weak and strong tetrel bonds? Electronic features of tetrahedral molecules interacted with halide anions

Ekaterina V. Bartashevich [a,*], Svetlana E. Mukhitdinova [a], Iliya V. Klyuev [a], Vladimir G. Tsirelson [a,b]

Table S1

Bond lengths and electron density at critical points (a. u.) for Tt–Y and Tt–Hal interactions in complexes $Y–TtX_3…Hal^-$

Name of complex	Tt–Y					Tt–X						Tt…Hal	
	Bond	Complex		Molecule		Bond	Complex		Molecule		Bond		
		r, Å	$\rho(r_{cp})$	r, Å	$\rho(r_{cp})$		r, Å	$\rho(r_{cp})$	r, Å	$\rho(r_{cp})$		r, Å	$\rho(r_{cp})$
$SiBr_3F...F^-$	Si–F	1,682	0,103	1,623	0,116	Si–Br	2,315	0,074	2,198	0,090	Si…F	2,224	0,073
$SiCl_3F..Cl^-$	Si–F	1,688	0,102	1,619	0,118	Si–Cl	2,142	0,080	2,038	0,097	Si…Cl	1,682	0,103
$SiBr_3Cl...Cl^-$	Si–Cl	2,226	0,073	2,051	0,094	Si–Br	2,310	0,076	2,208	0,089	Si…Cl	2,226	0,073
$SiBr_3F...Br^-$	Si–F	1,684	0,102	1,623	0,116	Si–Br	2,308	0,076	2,198	0,090	Si…Br	2,393	0,070
$SiBr_3Cl...Br^-$	Si–Cl	2,224	0,073	2,051	0,094	Si–Br	2,311	0,075	2,208	0,089	Si…Br	2,412	0,068
$SiBr_3CN...Br^-$	Si–C	1,929	0,094	1,845	0,108	Si–Br	2,306	0,077	2,203	0,089	Si…Br	2,390	0,071
$SiBr_3NO_2...Br^-$	Si–N	2,052	0,075	1,900	0,096	Si–Br	2,376	0,068	2,192	0,091	Si…Br	2,370	0,072
$SiBr_3CH_3...Br^-$	Si–C	1,914	0,110	1,855	0,119	Si–Br	2,313	0,075	2,227	0,085	Si…Br	2,390	0,070
$SiCl_3F...F^-$	Si–F	1,687	0,103	1,619	0,118	Si–Cl	2,149	0,079	2,038	0,097	Si…F	1,687	0,103
$SiCl_3Cl...Cl^-$	Si–Cl	2,134	0,082	2,048	0,095	Si–Cl	2,134	0,082	2,048	0,095	Si…Br	2,230	0,073
$SiCl_3Br...Br^-$	Si–Br	2,420	0,068	2,205	0,090	Si–Cl	2,130	0,083	2,049	0,095	Si…Br	2,420	0,068
$SiC_3H_9Cl...Cl^-$	Si–Cl	2,182	0,070	2,114	0,081	Si–C	1,870	0,114	1,875	0,113	Si…Cl	3,521	0,008
$SiC_3H_9F...F^-$	Si–F	1,796	0,077	1,663	0,104	Si–C	1,914	0,105	1,871	0,114	Si…F	1,809	0,075
$GeBr_3F...F^-$	Ge–F	1,771	0,132	1,714	0,150	Ge–Br	2,432	0,064	2,319	0,080	Ge…F	1,771	0,132
$GeF_3F...F^-$	Ge–F	1,752	0,135	1,693	0,158	Ge–F	1,752	0,135	1,693	0,158	Ge…F	1,775	0,129
$GeCl_3F...Cl^-$	Ge–F	1,688	0,129	1,714	0,150	Ge–Cl	2,203	0,093	2,108	0,116	Ge…Cl	2,224	0,077
$GeCl_3Cl...Cl^-$	Ge–Cl	2,201	0,094	2,118	0,113	Ge–Cl	2,201	0,094	2,118	0,113	Ge…Cl	2,308	0,077
$GeBr_3Cl...Cl^-$	Ge–Cl	2,280	0,081	2,116	0,113	Ge–Br	2,453	0,062	2,332	0,078	Ge…Cl	2,280	0,081
$GeBr_3F...Br^-$	Ge–F	1,765	0,133	1,714	0,150	Ge–Br	2,435	0,064	2,319	0,080	Ge…Br	2,572	0,052
$GeBr_3Br...Br^-$	Ge–Br	2,434	0,064	2,332	0,078	Ge–Br	2,434	0,064	2,332	0,078	Ge…Br	2,556	0,054
$GeBr_3Cl...Br^-$	Ge–Cl	2,260	0,084	2,116	0,113	Ge–Br	2,444	0,063	2,332	0,078	Ge…Br	2,583	0,051
$GeC_3H_9Cl...Cl^-$	Ge–Cl	2,598	0,040	2,190	0,093	Ge–C	1,943	0,131	1,934	0,134	Ge…Cl	2,503	0,047
$SnC_3H_9Cl...Cl^-$	Sn–Cl	2,661	0,039	2,371	0,076	Sn–C	2,073	0,118	2,070	0,119	Sn…Cl	2,662	0,039
$SnBr_3Cl...Cl^-$	Sn–Cl	2,380	0,078	2,263	0,100	Sn–Br	2,607	0,053	2,505	0,065	Sn…Cl	2,380	0,078
$SnBr_3Cl...Br^-$	Sn–Cl	2,366	0,080	2,263	0,100	Sn–Br	2,604	0,054	2,505	0,065	Sn…Br	2,652	0,050
$SnBr_3F...Br^-$	Sn–F	1,928	0,118	1,891	0,130	Sn–Br	2,592	0,054	2,494	0,066	Sn…Br	2,640	0,051
$SnCl_3Cl...Cl^-$	Sn–Cl	2,358	0,080	2,272	0,098	Sn–Cl	2,358	0,080	2,272	0,098	Sn…Cl	2,405	0,073
$SnCl_3F...Cl^-$	Sn–F	1,945	0,113	1,897	0,127	Sn–Cl	2,357	0,080	2,264	0,099	Sn…Cl	2,400	0,074
$PbBr_3F...Br^-$	Pb–F	2,041	0,100	2,005	0,109	Pb–Br	2,659	0,055	2,584	0,063	Pb…Br	2,700	0,052
$PbCl_3F...Cl^-$	Pb–F	2,045	0,099	2,002	0,110	Pb–Cl	2,458	0,072	2,386	0,084	Pb…Cl	2,489	0,068
$PbC_3H_9Cl...Cl^-$	Pb–Cl	2,655	0,047	2,461	0,071	Pb–C	2,236	0,095	2,226	0,097	Pb…Cl	2,655	0,047

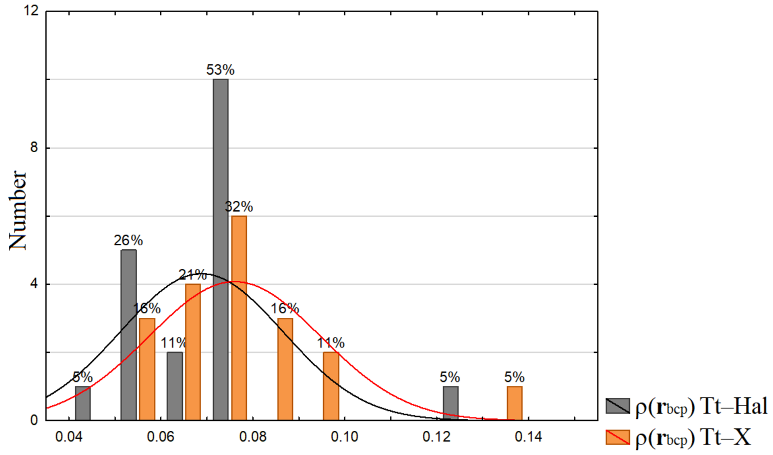

Figure S1. Histogram of electron density distribution at bcp for axial Hal⁻…Tt and equatorial Tt–X bonds of the same type, Hal⁻ = X, in series of complexes Y–TtHal$_3$…Hal⁻, Tt = Si, Ge, Sn, Pb.

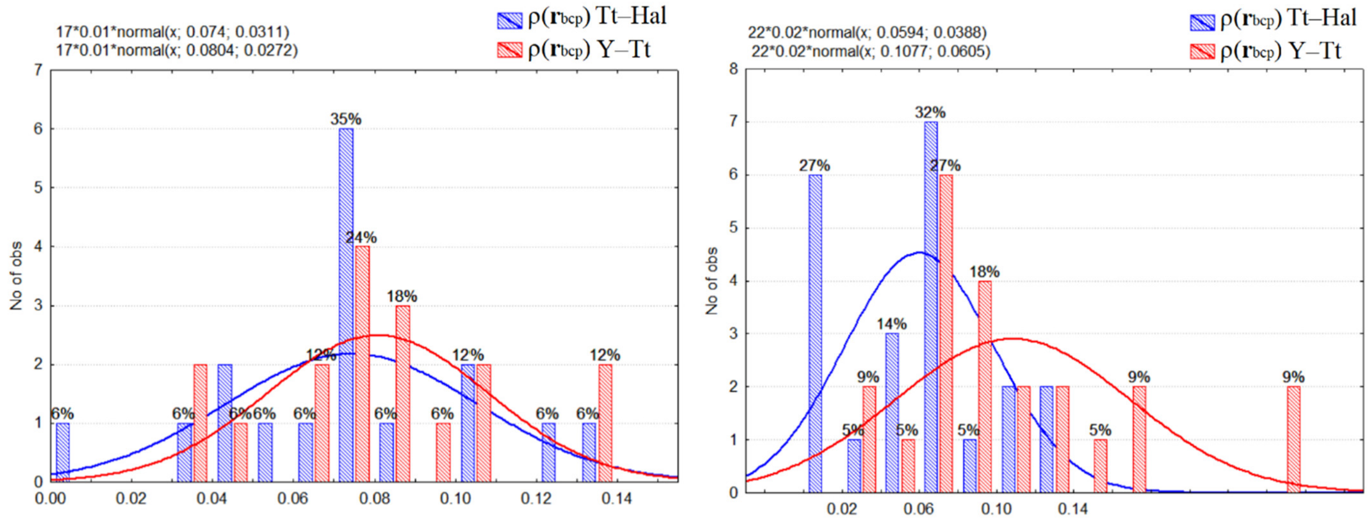

(a) (b)

Figure S2. Histogram of electron density distribution at bcp between axial Hal⁻…Tt and Y–Tt bonds of the same type, Hal⁻ = Y, in series of complexes Y–TtHal3…Hal⁻ a) Tt = Si, Ge, Sn, Pb; b) a) Tt = C, Si, Ge, Sn, Pb.

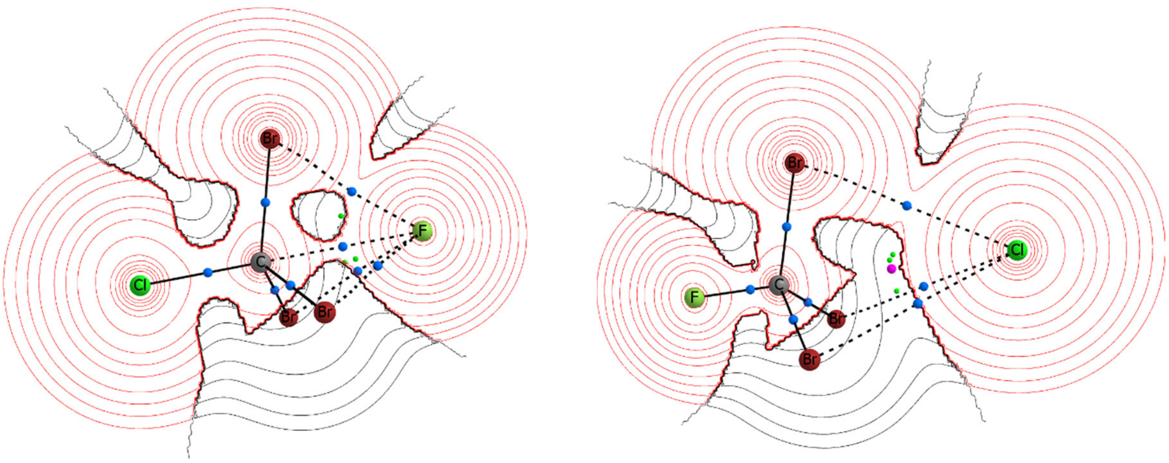

Figure S3. The distribution of sign[(λ_2(r)ρ(r)] function in the plane Br-C-Hal⁻ for complexes: a) Cl–CBr$_3$…F⁻ with bcp and b) F–CBr$_3$…Cl⁻ with ccp between Hal and Tt atoms.

Table S2

Properties of the static, electrostatic, and Pauli potentials, for Hal^-…Tt bonds in complexes $Y–TtX_3$…Hal^-

Complex	Bond	x_{es}, Å	$\upsilon_{es}(\mathbf{r})_{min}$, a.u.	x_{st}, Å	$\upsilon_{st}(\mathbf{r})_{max}$, a.u.	x_{ρ}, Å	$\rho(\mathbf{r})_{min}$, a.u.	$(x_{st} - x_{es}) = \Delta_{st\text{-}es}$, Å	$(x_{\rho} - x_{st}) = \Delta_{\rho\text{-}st}$, Å	$\upsilon_{xc}(\mathbf{r})_{ext}$, a.u.	$\upsilon_{P}(\mathbf{r})_{min1}$, a.u.	$\upsilon_{P}(\mathbf{r})_{min2}$, a.u.
1	2	3	4	5	6	7	8	9	10	11	12	13
CBr_3CN...F^-	C...F	1,759	-0,282	2,097	-0,282	2,154	0,016	0,338	0,056	-0,564	0,402	0,829
CBr_3CH_3...F^-	C...F	1,769	-0,324	2,147	-0,218	2,202	0,013	0,378	0,055	-0,542	0,350	0,728
CBr_3Cl...F^-	C...F	1,756	-0,257	2,041	-0,331	2,095	0,021	0,285	0,054	-0,588	0,455	0,827
CBr_3F...F^-	C...F	1,818	-0,293	2,165	-0,268	2,218	0,016	0,347	0,053	-0,561	0,382	0,834
CCl_3F...F^-	C...F	1,736	-0,303	2,063	-0,299	2,107	0,017	0,327	0,044	-0,601	0,348	1,018
CBr_3NO_2...F^-	C...F	1,744	-0,268	2,038	-0,332	2,094	0,022	0,295	0,056	-0,600	0,425	0,835
CBr_3NH_2...Cl^-	C...Cl	2,263	-0,269	2,722	-0,117	2,839	0,005	0,459	0,117	-0,386	0,184	0,666
CBr_3F...Cl^-	C...Cl	2,234	-0,243	2,640	-0,159	2,743	0,006	0,406	0,103	-0,402	0,216	0,679
CBr_3CN...Cl^-	C...Cl	2,229	-0,226	2,600	-0,189	2,703	0,007	0,371	0,102	-0,415	0,243	0,697
CBr_3Cl...Cl^-	C...Cl	2,242	-0,239	2,628	-0,165	2,729	0,006	0,386	0,101	-0,404	0,228	0,667
CBr_3CH_3...Cl^-	C...Cl	2,265	-0,267	2,708	-0,123	2,818	0,005	0,443	0,110	-0,390	0,184	0,629
CBr_3NH_2...Br^-	C...Br	2,404	-0,248	2,788	-0,126	2,926	0,005	0,384	0,138	-0,374	0,198	0,654
CBr_3Cl...Br^-	C...Cl	2,388	-0,221	2,723	-0,163	2,847	0,006	0,335	0,125	-0,384	0,250	0,667
CBr_3CN...Br^-	C...Cl	2,382	-0,210	2,704	-0,182	2,830	0,007	0,322	0,126	-0,392	0,261	0,693
CBr_3CH_3...Br^-	C...Br	2,402	-0,249	2,780	-0,128	2,917	0,005	0,378	0,137	-0,377	0,193	0,630
CBr_3F...Br^-	C...Br	2,380	-0,225	2,734	-0,157	2,857	0,006	0,353	0,123	-0,382	0,236	0,676
CCl_3F...Cl^-	C...Cl	2,242	-0,253	2,664	-0,151	2,818	0,005	0,422	0,154	-0,404	0,193	0,963
CCl_3F...Br^-	C...Br	2,366	-0,239	2,742	-0,151	2,926	0,005	0,376	0,184	-0,390	0,193	0,971
CCl_3Cl...Cl^-	C...Cl	2,242	-0,252	2,657	-0,153	2,811	0,005	0,415	0,154	-0,406	0,196	0,957
CCl_3Cl...Br^-	C...Br	2,362	-0,238	2,732	-0,155	2,917	0,005	0,370	0,185	-0,393	0,197	0,963
CCl_3Br...Br^-	C...Br	2,370	-0,234	2,720	-0,161	2,901	0,006	0,350	0,181	-0,395	0,204	0,951
$CC_3H_9NH_2$...Cl^-	C...Cl	2,293	-0,237	2,715	-0,160	2,888	0,005	0,423	0,173	-0,397	0,243	0,209
$CC_3H_9NO_2$...Cl^-	C...Cl	2,253	-0,205	2,657	-0,206	2,810	0,006	0,404	0,153	-0,410	0,275	1,102
CC_3H_9F...Cl^-	C...Cl	2,265	-0,224	2,686	-0,178	2,849	0,005	0,421	0,164	-0,402	0,247	1,166
CC_3H_9Cl...Cl^-	C...Cl	2,252	-0,214	2,667	-0,194	2,827	0,005	0,415	0,161	-0,407	0,263	1,125
CC_3H_9CN...Cl^-	C...Cl	2,255	-0,209	2,672	-0,198	2,837	0,005	0,417	0,165	-0,407	1,477	1,047
$CC_3H_9CH_3$...Cl^-	C...Cl	2,273	-0,240	2,720	-0,155	2,897	0,005	0,447	0,177	-0,395	0,231	1,003
$SiBr_3NO_2$...F^-	Si...F	1,508	0,589	1,570	-1,590	1,774	0,104	0,062	0,205	-1,001	1,268	5,538
$SiBr_3F$...F^-	Si...F	1,510	0,564	1,572	-1,563	1,777	0,103	0,062	0,205	-0,999	1,246	5,525
$SiBr_3CH_3$...F^-	Si...F	1,507	0,574	1,569	-1,575	1,773	0,105	0,062	0,203	-1,001	1,254	5,516
$SiCl_3F$...Cl^-	Si...Cl	1,868	0,270	1,913	-1,032	2,230	0,073	0,046	0,316	-0,762	0,600	-
$SiBr_3NO_2$...Cl^-	Si...Cl	1,853	0,319	1,900	-1,089	2,210	0,076	0,047	0,310	-0,770	0,647	-
$SiBr_3CN$...Cl^-	Si...Cl	1,862	0,300	1,907	-1,065	2,220	0,075	0,046	0,313	-0,765	0,635	-
$SiBr_3F$...Cl^-	Si...Cl	1,863	0,286	1,908	-1,049	2,223	0,074	0,046	0,314	-0,764	0,621	-
$SiBr_3Cl$...Cl^-	Si...Cl	1,869	0,279	1,915	-1,040	2,231	0,073	0,046	0,317	-0,760	0,621	-
$SiBr_3CH_3$...Cl^-	Si...Cl	1,860	0,290	1,906	-1,055	2,218	0,075	0,047	0,311	-0,765	0,626	-
$SiBr_3F$...Br^-	Si...Br	1,990	0,248	2,033	-0,945	2,340	0,070	0,042	0,308	-0,697	0,602	-
$SiBr_3Cl$...Br^-	Si...Br	2,000	0,242	2,041	-0,932	2,346	0,068	0,041	0,305	-0,690	0,606	-
$SiBr_3CN$...Br^-	Si...Br	1,997	0,264	2,031	-0,961	2,336	0,071	0,034	0,305	-0,697	0,617	-
$SiBr_3NO_2$...Br^-	Si...Br	1,987	0,283	2,022	-0,987	2,331	0,072	0,036	0,309	-0,703	0,626	-
$SiBr_3CH_3$...Br^-	Si...Br	1,997	0,251	2,031	-0,947	2,335	0,070	0,034	0,304	-0,696	0,605	-
$SiCl_3F$...F^-	Si...F	1,513	0,548	1,574	-1,545	1,781	0,103	0,061	0,207	-0,997	1,221	-
$SiCl_3CN$...Br^-	Si...Br	1,992	0,255	2,034	-0,951	2,341	0,070	0,042	0,307	-0,697	0,596	-
$SiCl_3F$...Br^-	Si...Br	1,997	0,232	2,040	-0,926	2,348	0,069	0,043	0,308	-0,694	0,578	-

$SiCl_3Br...Br^-$	Si…Br	2,004	0,237	2,045	-0,924	2,348	0,068	0,041	0,303	-0,687	0,594	-
$SiCl_3NO_2...Br^-$	Si...Br	1,990	0,274	2,026	-0,977	2,335	0,072	0,036	0,309	-0,703	0,607	-
$SiCl_3CH_3...Br^-$	Si...Br	2,001	0,224	2,044	-0,914	2,348	0,068	0,043	0,304	-0,689	0,574	-
$SiCl_3Cl...Cl^-$	Si...Cl	1,871	0,271	1,917	-1,031	2,233	0,073	0,046	0,317	-0,760	0,605	-
$SiCl_3Cl...Br^-$	Si…Br	2,004	0,232	2,045	-0,920	2,351	0,068	0,041	0,306	-0,688	0,585	-
$SiC_3H_9CH_3...Cl^-$	Si...Cl	2,282	-0,234	2,699	-0,164	2,824	0,005	0,417	0,125	-0,398	0,226	0,749
$SiC_3H_9Cl...Cl^-$	Si...Cl	2,227	-0,196	2,557	-0,241	2,699	0,008	0,330	0,141	-0,437	0,264	0,778
$SiC_3H_9NO_2...Cl^-$	Si...Cl	1,924	0,123	2,000	-0,817	2,318	0,051	0,076	0,318	-0,694	0,519	-
$SiC_3H_9CN...Cl^-$	Si...Cl	2,217	-0,188	2,536	-0,256	2,674	0,008	0,319	0,138	-0,444	0,274	0,766
$SiC_3H_9NH_2...Cl^-$	Si...Cl	2,276	-0,232	2,694	-0,168	2,824	0,005	0,417	0,130	-0,400	0,227	0,784
$SiC_3H_9F...Cl^-$	Si...Cl	2,242	-0,211	2,608	-0,212	2,747	0,006	0,367	0,139	-0,423	0,247	0,806
$SiC_3H_9Cl...F^-$	Si...Cl	1,533	0,416	1,604	-1,380	1,819	0,086	0,071	0,215	-0,963	0,426	-
$SiC_3H_9Cl...Br^-$	Si...Br	2,377	-0,189	2,676	-0,223	2,847	0,007	0,299	0,171	-0,412	0,254	0,778
$SiC_3H_9CN...Br^-$	Si...Br	2,365	-0,180	2,653	-0,237	2,820	0,008	0,288	0,167	-0,417	0,269	0,764
$SiC_3H_9F...Br^-$	Si...Br	2,385	-0,202	2,718	-0,199	2,889	0,006	0,333	0,172	-0,401	0,232	0,805
$SiC_3H_9NH_2...Br^-$	Si...Br	2,422	-0,224	2,794	-0,160	2,960	0,005	0,372	0,167	-0,383	0,200	0,780
$SiC_3H_9CH_3...Br^-$	Si...Br	2,427	-0,225	2,800	-0,157	2,961	0,005	0,373	0,161	-0,382	0,198	0,744
$SiC_3H_9CH_3...F^-$	Si...F	1,882	-0,276	2,609	-0,272	2,775	0,012	0,726	0,166	-0,548	0,380	0,859
$SiC_3H_9NH_2...F^-$	Si...Br	1,442	0,239	1,603	-1,161	2,052	0,072	0,161	0,448	-0,922	0,381	0,885
$SiC_3H_9CN...F^-$	Si...F	1,541	0,350	1,617	-1,300	1,841	0,081	0,076	0,224	-0,950	1,001	-
$SiC_3H_9F...F^-$	Si...F	1,549	0,282	1,631	-1,216	1,862	0,075	0,083	0,231	-0,934	0,929	-
$GeBr_3CN...F^-$	Ge...F	1,538	0,519	1,592	-1,480	1,716	0,132	0,054	0,124	-0,961	1,387	-
$GeBr_3NO_2...F^-$	Ge...F	1,535	0,529	1,588	-1,495	1,712	0,133	0,054	0,124	-0,966	1,390	-
$GeBr_3F...F^-$	Ge...F	1,532	0,508	1,592	-1,470	1,716	0,132	0,060	0,124	-0,961	1,376	-
$GeF4...F^-$	Ge...F	1,534	0,455	1,595	-1,419	1,721	0,129	0,062	0,126	-0,964	1,293	-
$GeCl_3F...Cl^-$	Ge...Cl	1,884	0,286	1,927	-1,024	2,065	0,079	0,043	0,138	-0,737	0,719	-
$GeCl4...Cl^-$	Ge...Cl	1,886	0,277	1,936	-1,007	2,073	0,077	0,051	0,136	-0,730	0,716	-
$GeCl_2F_2...Cl^-$	Ge...Cl	1,884	0,282	1,928	-1,018	2,064	0,079	0,044	0,136	-0,736	0,744	-
$GeF4...Cl^-$	Ge...Cl	1,875	0,282	1,920	-1,026	2,061	0,081	0,045	0,140	-0,744	0,689	-
$GeBr_3F...Cl^-$	Ge...Cl	1,876	0,317	1,919	-1,056	2,047	0,082	0,042	0,129	-0,739	0,767	-
$GeBr_3Cl...Cl^-$	Ge...Cl	1,879	0,313	1,922	-1,050	2,052	0,081	0,043	0,130	-0,737	0,759	-
$GeBr_3F...Br^-$	Ge...Br	2,063	0,226	2,102	-0,849	2,172	0,052	0,039	0,069	-0,623	0,827	-
$GeBr4...Br^-$	Ge...Br	2,055	0,242	2,095	-0,872	2,169	0,054	0,040	0,073	-0,629	0,833	-
$GeCl4...Br^-$	Ge...Br	2,079	0,179	2,127	-0,795	2,214	0,048	0,048	0,087	-0,616	0,744	-
$GeF4...Br^-$	Ge...Br	2,043	0,215	2,094	-0,846	2,187	0,052	0,051	0,092	-0,631	0,761	-
$GeBr_3Cl...Br^-$	Ge...Br	2,068	0,218	2,107	-0,840	2,180	0,051	0,039	0,072	-0,622	0,811	-
$GeCl_3NO_2...Br^-$	Ge...Br	2,060	0,227	2,100	-0,855	2,183	0,053	0,040	0,083	-0,628	0,799	-
$GeC_3H_9CN...Cl^-$	Ge...Cl	2,181	-0,167	2,443	-0,306	2,572	0,012	0,262	0,129	-0,472	0,296	0,809
$GeC_3H_9F...Cl^-$	Ge...Cl	2,160	-0,161	2,399	-0,327	2,540	0,013	0,238	0,141	-0,488	0,301	-
$GeC_3H_9CH_3...Cl^-$	Ge...Cl	2,272	-0,229	2,660	-0,177	2,783	0,006	0,388	0,123	-0,406	0,231	0,709
$GeC_3H_9NH_2...Cl^-$	Ge...Cl	2,252	-0,223	2,629	-0,192	2,758	0,006	0,377	0,129	-0,415	0,236	0,736
$GeC_3H_9NO_2...Cl^-$	Ge...Cl	1,976	0,073	2,062	-0,713	2,202	0,044	0,087	0,139	-0,640	0,562	-
$GeC_3H_9Cl...Cl^-$	Ge...Cl	1,992	0,045	2,090	-0,671	2,231	0,040	0,098	0,141	-0,626	0,580	-
$GeC_3H_9Cl...Br^-$	Ge...Br	2,174	-0,029	2,288	-0,512	2,419	0,027	0,114	0,131	-0,541	0,474	-
$SnCl4...Br^-$	Sn...Br	2,071	0,247	2,112	-0,861	2,200	0,048	0,041	0,088	-0,613	0,821	-
$SnI_2F_2...Cl^-$	Sn...Cl	1,889	0,316	1,932	-1,044	2,046	0,077	0,042	0,114	-0,728	0,757	-
$SnC_3H_9F...Cl^-$	Sn...Cl	2,017	0,009	2,136	-0,604	2,271	0,031	0,119	0,135	-0,594	0,502	-
$SnC_3H_9CH_3...Cl^-$	Sn...Cl	2,158	-0,162	2,392	-0,324	2,522	0,012	0,234	0,130	-0,485	0,313	0,841
$SnC_3H_9Cl...Cl^-$	Sn...Cl	1,980	0,084	2,076	-0,712	2,212	0,039	0,096	0,136	-0,628	0,568	-
$SnC_3H_9CN...Cl^-$	Sn...Cl	2,031	-0,003	2,159	-0,580	2,294	0,029	0,128	0,135	-0,583	0,492	-
$SnBr_3Cl...Cl^-$	Sn...Cl	1,889	0,331	1,928	-1,060	2,044	0,078	0,040	0,115	-0,730	0,775	-

SnBr$_3$Cl...F$^-$	Sn...Cl	1,887	0,331	1,928	-1,061	2,040	0,078	0,041	0,112	-0,730	0,778	-
SnBr$_3$Cl...Br$^-$	Sn...Br	2,061	0,279	2,100	-0,896	2,178	0,050	0,040	0,078	-0,617	0,875	-
SnBr$_3$F...Br$^-$	Sn...Br	2,063	0,284	2,096	-0,904	2,170	0,051	0,032	0,075	-0,620	0,881	-
SnC$_3$H$_9$NH$_2$...Cl$^-$	Sn...Cl	2,103	-0,110	2,290	-0,414	2,422	0,018	0,187	0,132	-0,524	0,376	-
SnC$_3$H$_9$NO$_2$...Cl$^-$	Sn...Cl	1,980	0,085	2,076	-0,714	2,212	0,040	0,096	0,136	-0,629	0,569	-
SnCl$_3$F...Br$^-$	Sn...Br	2,064	0,251	2,107	-0,868	2,191	0,049	0,042	0,085	-0,617	0,826	-
SnCl$_3$Cl...Cl$^-$	Sn...Cl	1,892	0,295	1,942	-1,016	2,061	0,073	0,049	0,120	-0,722	0,733	-
SnCl$_3$F...Cl$^-$	Sn...Cl	1,898	0,294	1,941	-1,017	2,058	0,074	0,043	0,117	-0,723	0,734	-
PbBr$_3$CN...F$^-$	Pb...F	1,697	0,305	1,733	-1,077	1,867	0,089	0,036	0,134	-0,772	1,305	-
PbBr$_3$F...Cl$^-$	Pb...Cl	1,898	0,293	1,952	-0,999	2,061	0,069	0,054	0,109	-0,706	0,753	-
PbBr$_3$F...Br$^-$	Pb...Br	2,058	0,266	2,097	-0,890	2,174	0,052	0,039	0,077	-0,624	0,824	-
PbCl$_3$F...Cl$^-$	Pb...Cl	1,900	0,283	1,954	-0,988	2,064	0,068	0,054	0,110	-0,706	0,734	-
PbCl$_3$Cl...Br$^-$	Pb...Br	2,054	0,255	2,100	-0,878	2,183	0,051	0,046	0,083	-0,623	0,799	-
PbC$_3$H$_9$NH$_2$...Cl$^-$	Pb...Cl	1,993	0,031	2,100	-0,645	2,212	0,039	0,107	0,112	-0,614	0,516	-
PbC$_3$H$_9$NO$_2$...Cl$^-$	Pb...Cl	1,947	0,139	2,030	-0,796	2,141	0,050	0,083	0,111	-0,656	0,607	-
PbC$_3$H$_9$CH$_3$...Cl$^-$	Pb...Cl	2,016	0,003	2,127	-0,603	2,240	0,035	0,110	0,113	-0,600	0,493	-
PbC$_3$H$_9$CN...Cl$^-$	Pb...Cl	1,971	0,098	2,056	-0,739	2,167	0,046	0,085	0,111	-0,640	0,574	-
PbC$_3$H$_9$Cl...Cl$^-$	Pb...Cl	1,960	0,115	2,045	-0,763	2,158	0,047	0,085	0,113	-0,648	0,584	-
PbC$_3$H$_9$F...Cl$^-$	Pb...Cl	1,975	0,084	2,062	-0,721	2,173	0,045	0,087	0,111	-0,637	0,560	-

Table S3

The static, electrostatic potentials, exchange-correlation contribution in total static potential at the critical points of electron density and bond contribution to exchange energy for $Hal^-\ldots Tt$ bonds in complexes $Y–TtX_3\ldots Hal^-$

Complexes	Bond	$\upsilon_{es}(\mathbf{r}_{cp})$, a.u.	$\upsilon_{st}(\mathbf{r}_{cp})$, a.u.	$\upsilon_{ex}(\mathbf{r}_{cp})$, a.u.	Vx, a.u.	$\rho(\mathbf{r}_{cp})$, a.u.	r, Å	E_{bind}, kcal/mol
1	2	4	6	7	5	3	8	9
CBr$_3$CN...F$^-$	C...F	-0,186	-0,287	-0,473	-0,018	0,016	2,746	-11,6
CBr$_3$CH$_3$...F$^-$	C...F	-0,227	-0,223	-0,450	-0,015	0,013	2,879	1,3
CBr$_3$Cl...F$^-$	C...F	-0,172	-0,336	-0,509	-0,027	0,021	2,614	-9,5
CBr$_3$F...F$^-$	C...F	-0,194	-0,274	-0,468	-0,017	0,016	2,741	-5,4
CCl$_3$F...F$^-$	C...F	-0,180	-0,311	-0,491	-0,010	0,017	2,667	-5,2
CBr$_3$NO$_2$...F$^-$	C...F	-0,172	-0,338	-0,510	-0,022	0,022	2,604	-11,2
CBr$_3$NH$_2$...Cl$^-$	C...Cl	-0,212	-	-	-	0,004	3,987	3,8
CBr$_3$F...Cl$^-$	C...Cl	-0,183	-	-	-	0,006	3,732	-1,6
CBr$_3$CN...Cl$^-$	C...Cl	-0,169	-	-	-	0,007	3,620	-5,8
CBr$_3$Cl...Cl$^-$	C...Cl	-0,184	-	-	-	0,006	3,725	-1,6
CBr$_3$CH$_3$...Cl$^-$	C...Cl	-0,206	-	-	-	0,005	3,910	3,7
CBr$_3$NH$_2$...Br$^-$	C...Br	-0,198	-	-	-	0,005	4,003	4,4
CBr$_3$Cl...Br$^-$	C...Cl	-0,175	-	-	-	0,006	3,840	-1,0
CBr$_3$CN...Br$^-$	C...Cl	-0,162	-	-	-	0,007	3,752	-5,0
CBr$_3$CH$_3$...Br$^-$	C...Br	-0,197	-	-	-	0,005	3,972	4,3
CBr$_3$F...Br$^-$	C...Br	-0,174	-	-	-	0,006	3,847	-0,6
CCl$_3$F...Cl$^-$	C...Cl	-0,177	-	-	-	0,005	3,776	-2,1
CCl$_3$F...Br$^-$	C...Br	-0,169	-	-	-	0,005	3,864	-0,9
CCl$_3$Cl...Cl$^-$	C...Cl	-0,178	-	-	-	0,005	3,777	-2,1
CCl$_3$Cl...Br$^-$	C...Br	-0,170	-	-	-	0,005	3,856	-0,9
CCl$_3$Br...Br$^-$	C...Br	-0,169	-	-	-	0,006	3,825	-1,2
CC$_3$H$_9$NH$_2$...Cl$^-$	C...Cl	-0,186	-	-	-	0,005	3,923	-9,1
CC$_3$H$_9$NO$_2$...Cl$^-$	C...Cl	-0,154	-	-	-	0,006	3,751	-19,2

$CC_3H_9F...Cl^-$	C...Cl	-0,171	-	-	-	0,005	3,805	-13,7
$CC_3H_9Cl...Cl^-$	C...Cl	-0,162	-	-	-	0,005	3,774	-15,9
$CC_3H_9CN...Cl^-$	C...Cl	-0,158	-	-	-	0,005	3,807	-17,7
$CC_3H_9CH_3...Cl^-$	C...Cl	-0,189	-	-	-	0,005	3,956	-8,0
$SiBr_3NO_2...F^-$	Si...F	1,129	-1,951	-0,823	-0,114	0,104	1,676	-132,4
$SiBr_3F...F^-$	Si...F	1,105	-1,941	-0,836	-0,112	0,103	1,682	-121,4
$SiBr_3CH_3...F^-$	Si...F	1,111	-1,951	-0,840	-0,117	0,105	1,675	-114,8
$SiCl_3F...Cl^-$	Si...Cl	0,782	-1,444	-0,662	-0,080	0,073	2,224	-72,2
$SiBr_3NO_2...Cl^-$	Si...Cl	0,833	-1,509	-0,675	-0,099	0,076	2,195	-83,4
$SiBr_3CN...Cl^-$	Si...Cl	0,809	-1,478	-0,669	-0,097	0,075	2,212	-77,8
$SiBr_3F...Cl^-$	Si...Cl	0,796	-1,463	-0.667	-0,096	0,074	2,214	-71,8
$SiBr_3Cl...Cl^-$	Si...Cl	0,790	-1,452	-0,662	-0,096	0,073	2,226	-71,3
$SiBr_3CH_3...Cl^-$	Si...Cl	0,798	-1,468	-0,670	-0,102	0,075	2,208	-66,0
$SiBr_3F...Br^-$	Si...Br	0,636	-1,264	-0,628	-0,107	0,070	2,393	-64,5
$SiBr_3Cl...Br^-$	Si...Br	0,612	-1,233	-0,621	-0,107	0,068	2,412	-63,4
$SiBr_3CN...Br^-$	Si...Br	0,646	-1,274	-0,628	-0,109	0,071	2,390	-70,3
$SiBr_3NO_2...Br^-$	Si...Br	0,689	-1,323	-0,634	-0,110	0,072	2,370	-75,9
$SiBr_3CH_3...Br^-$	Si...Br	0,630	-1,258	-0,628	-0,113	0,070	2,390	-58,2
$SiCl_3F...F^-$	Si...F	1,092	-1,924	-0,832	-0,095	0,103	1,687	-123,7
$SiCl_3CN...Br^-$	Si...Br	0,641	-1,274	-0,633	-0,092	0,070	2,396	-73,7
$SiCl_3F...Br^-$	Si...Br	0,616	-1,238	-0,621	-0,089	0,069	2,407	-65,5
$SiCl_3Br...Br^-$	Si...Br	0,603	-1,220	-0,616	-0,090	0,068	2,420	-67,7
$SiCl_3NO_2...Br^-$	Si...Br	0,680	-1,311	-0,631	-0,092	0,072	2,376	-79,3
$SiCl_3CH_3...Br^-$	Si...Br	0,595	-1,214	-0,619	-0,093	0,068	2,415	-58,2
$SiCl_3Cl...Cl^-$	Si...Cl	0,784	-1,443	-0,660	-0,079	0,073	2,230	-74,4
$SiCl_3Cl...Br^-$	Si…Br	0,602	-1,219	-0,617	-0,089	0,068	2,420	-66,3
$SiC_3H_9CH_3...Cl^-$	Si...Cl	-0,170	-	-	-	0,005	3,847	-9,9
$SiC_3H_9Cl...Cl^-$	Si...Cl	-0,115	-0,257	-0,372	-0,002	0,008	3,521	-18,7
$SiC_3H_9NO_2...Cl^-$	Si...Cl	0,545	-1,127	-0,582	-0,054	0,051	2,389	-52,1
$SiC_3H_9CN...Cl^-$	Si...Cl	-0,106	-0,272	-0,378	-0,003	0,008	3,474	-21,3
$SiC_3H_9NH_2...Cl^-$	Si...Cl	-0,166	-	-	-	0,005	3,832	-10,2
$SiC_3H_9F...Cl^-$	Si...Cl	-0,134	-	-	-	0,006	3,626	-15,6
$SiC_3H_9Cl...F^-$	Si...Cl	0,178	-0,693	-0,515	-0,030	0,034	2,655	-105,8
$SiC_3H_9Cl...Br^-$	Si...Br	-0,115	-0,244	-0,358	-0,002	0,007	3,694	-17,4
$SiC_3H_9CN...Br^-$	Si...Br	-0,107	-0,258	-0,364	-0,002	0,008	3,645	-20,0
$SiC_3H_9F...Br^-$	Si...Br	-0,133	-	-	-	0,006	3,783	-14,6
$SiC_3H_9NH_2...Br^-$	Si...Br	-0,162	-	-	-	0,005	3,967	-9,5
$SiC_3H_9CH_3...Br^-$	Si...Br	-0,166	-	-	-	0,005	3,979	-9,2
$SiC_3H_9CH_3...F^-$	Si...F	-0,156	-	-	-	0,012	2,964	-22,0
$SiC_3H_9NH_2...F^-$	Si...Br	-0,140	-	-	-	0,013	1,835	-59,6
$SiC_3H_9CN...F^-$	Si...F	0,891	-1,659	-0,768	-0,073	0,081	1,777	-87,8
$SiC_3H_9F...F^-$	Si...F	0,826	-1,573	-0,748	-0,064	0,075	1,809	-74,0
$GeBr_3CN...F^-$	Ge...F	0,748	-1,615	-0,867	-0,171	0,132	1,770	-117,5
$GeBr_3NO_2...F^-$	Ge...F	0,761	-1,632	-0,871	-0,173	0,133	1,770	-125,0
$GeBr_3F...F^-$	Ge...F	0,737	-1,604	-0,867	-0,169	0,132	1,771	-103,7
$GeF4...F^-$	Ge...F	0,695	-1,558	-0,863	-0,136	0,129	1,775	-136,6
$GeCl_3F...Cl^-$	Ge...Cl	0,386	-1,074	-0,688	-0,125	0,077	2,224	-71,3
$GeCl4...Cl^-$	Ge...Cl	0,390	-1,076	-0,686	-0,120	0,077	2,308	-72,3
$GeCl_2F_2...Cl^-$	Ge...Cl	0,398	-1,090	-0,693	-0,119	0,079	2,279	-76,0
$GeF4...Cl^-$	Ge...Cl	0,409	-1,106	-0,697	-0,112	0,081	2,271	-85,8
$GeBr_3F...Cl^-$	Ge...Cl	0,419	-1,121	-0,702	-0,140	0,082	2,273	-73,0

$GeBr_3Cl\ldots Cl^-$	Ge...Cl	0,418	-1,117	-0,699	-0,139	0,081	2,280	-72,2
$GeBr_3F\ldots Br^-$	Ge...Br	0,254	-0,862	-0,608	-0,118	0,052	2,572	-64,5
$GeBr4\ldots Br^-$	Ge...Br	0,273	-0,886	-0,613	-0,124	0,054	2,556	-67,6
$GeCl4\ldots Br^-$	Ge...Br	0,220	-0,813	-0,594	-0,100	0,048	2,621	-60,9
$GeF4\ldots Br^-$	Ge...Br	0,263	-0,869	-0,606	-0,098	0,052	2,548	-74,6
$GeBr_3Cl\ldots Br^-$	Ge...Br	0,247	-0,854	-0,607	-0,118	0,051	2,583	-63,0
$GeCl_3NO_2\ldots Br^-$	Ge...Br	0,265	-0,873	-0,609	-0,111	0,053	2,566	-71,6
$GeC_3H_9CN\ldots Cl^-$	Ge...Cl	-0,084	-0,323	-0,407	-0,010	0,012	3,302	-23,3
$GeC_3H_9F\ldots Cl^-$	Ge...Cl	-0,072	-0,349	-0,420	-0,013	0,013	3,213	-20,6
$GeC_3H_9CH_3\ldots Cl^-$	Ge...Cl	-0,162	-0,188	-0,350	-0,002	0,006	3,771	-10,6
$GeC_3H_9NH_2\ldots Cl^-$	Ge...Cl	-0,151	-0,205	-0,356	-0,003	0,006	3,699	-11,9
$GeC_3H_9NO_2\ldots Cl^-$	Ge...Cl	0,181	-0,762	-0,582	-0,069	0,044	2,546	-45,5
$GeC_3H_9Cl\ldots Cl^-$	Ge...Cl	0,210	-0,717	-0,580	-0,076	0,047	2,503	-41,4
$GeC_3H_9Cl\ldots Br^-$	Ge...Br	0,043	0,540	-0,496	-0,043	0,027	3,002	-32,0
$SnCl4\ldots Br^-$	Sn...Br	0,286	-0,880	-0,594	-0,103	0,048	2,673	-70,0
$SnI_2F_2\ldots Cl^-$	Sn...Cl	0,397	-1,096	-0,698	-0,126	0,077	2,382	-77,9
$SnC_3H_9F\ldots Cl^-$	Sn...Cl	0,106	-0,643	-0,537	-0,050	0,031	2,778	-35,5
$SnC_3H_9CH_3\ldots Cl^-$	Sn...Cl	-0,077	-0,343	-0,421	-0,015	0,012	3,295	-15,5
$SnC_3H_9Cl\ldots Cl^-$	Sn...Cl	0,184	-0,758	-0,575	-0,066	0,039	2,662	-47,7
$SnC_3H_9CN\ldots Cl^-$	Sn...Cl	0,091	-0,616	-0,525	-0,045	0,029	2,827	-36,9
$SnBr_3Cl\ldots Cl^-$	Sn...Cl	0,413	-1,113	-0,700	-0,132	0,078	2,380	-80,3
$SnBr_3Cl\ldots F^-$	Sn...Cl	0,410	-1,112	-0,702	-0,132	0,078	2,377	-81,2
$SnBr_3Cl\ldots Br^-$	Sn...Br	0,310	-0,912	-0,602	-0,114	0,050	2,652	-71,8
$SnBr_3F\ldots Br^-$	Sn...Br	0,312	-0,919	-0,607	-0,116	0,051	2,640	-73,5
$SnC_3H_9NH_2\ldots Cl^-$	Sn...Cl	-0,022	-0,439	-0,460	-0,024	0,018	3,085	-20,3
$SnC_3H_9NO_2\ldots Cl^-$	Sn...Cl	0,184	-0,760	-0,576	-0,067	0,040	2,661	-48,1
$SnCl_3F\ldots Br^-$	Sn...Br	0,288	-0,887	-0,599	-0,106	0,049	2,659	-71,0
$SnCl_3Cl\ldots Cl^-$	Sn...Cl	0,384	-1,071	-0,688	-0,118	0,073	2,405	-79,4
$SnCl_3F\ldots Cl^-$	Sn...Cl	0,379	-1,069	-0,690	-0,120	0,074	2,400	-79,7
$PbBr_3CN\ldots F^-$	Pb...F	0,519	-1,320	-0,801	-0,138	0,100	2,044	-119,5
$PbBr_3F\ldots Cl^-$	Pb...Cl	0,370	-1,044	-0,674	-0,124	0,069	2,486	-84,5
$PbBr_3F\ldots Br^-$	Pb...Br	0,300	-0,907	-0,607	-0,120	0,052	2,700	-75,2
$PbCl_3F\ldots Cl^-$	Pb...Cl	0,363	-1,034	-0,672	-0,118	0,068	2,489	-86,5
$PbCl_3Cl\ldots Br^-$	$Pb\ldots Br^-$	0,294	-0,897	-0,604	-0,113	0,051	2,709	-87,3
$PbC_3H_9NH_2\ldots Cl^-$	Pb...Cl	0,113	-0,676	-0,564	-0,060	0,039	2,764	-31,6
$PbC_3H_9NO_2\ldots Cl^-$	Pb...Cl	0,222	-0,834	-0,612	-0,085	0,050	2,626	-55,6
$PbC_3H_9CH_3\ldots Cl^-$	Pb...Cl	0,085	-0,632	-0,547	-0,054	0,035	2,815	-27,2
$PbC_3H_9CN\ldots Cl^-$	Pb...Cl	0,181	-0,774	-0,593	-0,074	0,046	2,678	-47,6
$PbC_3H_9Cl\ldots Cl^-$	Pb...Cl	0,200	-0,800	-0,601	-0,079	0,047	2,655	-50,6
$PbC_3H_9F\ldots Cl^-$	Pb...Cl	0,166	-0,756	-0,589	-0,073	0,045	2,687	-43,8
